# Zebrafish VCAP1X2 regulates cardiac contractility and proliferation of cardiomyocytes and epicardial cells

**DOI:** 10.1038/s41598-018-26110-3

**Published:** 2018-05-18

**Authors:** Fang-Chi Hsieh, Yu-Fen Lu, Ian Liau, Chien-Chang Chen, Chao-Min Cheng, Chung-Der Hsiao, Sheng-Ping L. Hwang

**Affiliations:** 1 Graduate Institute of Life Sciences, National Defense Medical Center, Taipei, 11490 Taiwan; 2 Institute of Cellular and Organismic Biology, Academia Sinica, Taipei, 11529 Taiwan; 3 Department of Applied Chemistry and Institute of Molecular Science, National Chiao Tung University, Hsinchu, 30010 Taiwan; 4 Institue of Biomedical Sciences, Academia Sinica, Taipei, 11529 Taiwan; 5 Institute of Nanoengineering and Microsystems, National Tsing Hua University, Hsinchu, 30013 Taiwan; 6 Department of Bioscience Technology, Chung Yuan Christian University, Taoyuan, 32023 Taiwan

## Abstract

Sarcomeric signaling complexes are important to sustain proper sarcomere structure and function, however, the mechanisms underlying these processes are not fully elucidated. In a gene trap experiment, we found that *vascular cell adhesion protein 1 isoform X2* (*VCAP1X2)* mutant embryos displayed a dilated cardiomyopathy phenotype, including reduced cardiac contractility, enlarged ventricular chamber and thinned ventricular compact layer. Cardiomyocyte and epicardial cell proliferation was decreased in the mutant heart ventricle, as was the expression of pAKT and pERK. Contractile dysfunction in the mutant was caused by sarcomeric disorganization, including sparse myofilament, blurred Z-disc, and decreased gene expression for sarcomere modulators (*smyd1b*, *mypn* and *fhl2a*), sarcomeric proteins (*myh6*, *myh7*, *vmhcl* and *tnnt2a*) and calcium regulators (*ryr2b* and *slc8a1a*). Treatment of PI3K activator restored Z-disc alignment while injection of *smyd1b* mRNA restored Z-disc alignment, contractile function and cardiomyocyte proliferation in ventricles of *VCAP1X2* mutant embryos. Furthermore, injection of *VCAP1X2* variant mRNA rescued all phenotypes, so long as two cytosolic tyrosines were left intact. Our results reveal two tyrosine residues located in the VCAP1X2 cytoplasmic domain are essential to regulate cardiac contractility and the proliferation of ventricular cardiomyocytes and epicardial cells through modulating pAKT and pERK expression levels.

## Introduction

The sarcomere is the basic contractile unit of cardiac and skeletal striated muscles^1^, consisting of central bipolar myosin thick filaments surrounded by parallel actin thin filaments, which are anchored to the Z-disc. Mutations in thin or thick filament components may cause hypertrophic cardiomyopathy or dilated cardiomyopathy (DCM)^2,3^. In addition to well-known calcium homeostasis and cell signaling events, cardiac contractility is regulated by sarcomere maintenance. The sarcomere is a dynamic structure under constant turnover, and the homeostatic quality control system involves chaperones, the calpain and ubiquitin proteasome system, and autophagy^4^. In addition to protein quality control, other modulators participate in the maintenance of sarcomere homeostasis in cardiac and skeletal muscle. For instance, Smyd1, a MYND and SET-domain-containing lysine methyltransferase, is specifically expressed in muscle tissues. *Smyd1* knockout mice display defects in ventricular cardiomyocyte maturation and formation of the right ventricle^5^, and the protein localizes to the sarcomere of murine cardiomyocytes where it interacts with myosin at the sarcomeric M-line^6^. Furthermore, *fla/smyd1* mutant zebrafish exhibited disrupted sarcomere assembly in both heart and skeletal muscle, demonstrating the importance of the Smyd1-myosin interaction in the assembly of thick filaments^6^.

Z-discs contain α-actinin as a core protein^7^, which interacts with F-actin and titin. These interactions are critical for mechanical function. In addition to its mechanical role, Z-disc proteins are important for cell signaling, gene expression and cell survival^8^, with mutations in α-actinin, calsarin, telethonin or MLP all causing DCM or hypertrophic cardiomyopathy^3^. The Z-disc is linked to the sarcolemma via desmin intermediate filaments, which connect to costameres^7^. Two major protein complexes, including the dystrophin-glycoprotein complex (DGC) and the integrin-vinculin-talin complex, exist in the costamere^9^. Mutations in several proteins that comprise the DGC may cause cardiomyopathy or muscular dystrophy^10^. The integrin heterodimer transduces signals through focal adhesion kinase or integrin-linked kinase (ILK) to regulate cardiac growth, contractility and repair^11^. Cardiac deletion of *vinculin*, *β1 integrin* or *ILK* in mice led to dilated cardiomyopathy^3^. Therefore, the costamere-Z-disc axis is important for force and signal transmission between the sarcomere, sarcolemma and extracellular matrix. Further characterization of proteins within this axis will serve to advance our knowledge of mechanotransduction in cardiac muscle.

Recently, the zebrafish has emerged as a model organism for cardiovascular research, partially due to the availability of the zebrafish genome sequence and conserved function of many human and zebrafish genes. Zebrafish heart development and function resemble those of mammals, allowing researchers to use genetic tools for studies on cardiogenesis or cardiac diseases. Intriguingly, zebrafish also have the ability to regenerate heart muscle, suggesting that this model may hold clues to uncovering therapeutic treatments for heart failure^12,13^. In order to discover proteins that modulate cardiac development, we conducted a Tol2 transposon-mediated gene trap study and identified a *vascular cell adhesion protein 1 isoform X2* (*VCAP1X2)* mutant that displayed a DCM phenotype. A BLAST search revealed that the mammalian homologue to VCAP1X2 is vascular cell adhesion molecule 1 (VCAM1). Mammalian VCAM1 has been implicated in leukocyte transendothelial migration via engagement of the cell-surface ligand, very late antigen 4 (VLA4/α_4_β_1_-integrin)^14,15^. Moreover, *VCAM1*-deficient mouse embryonic hearts displayed thinned compact layer, reduction of intraventricular septum and epicardium formation defects, and the interaction between VCAM1 and integrin α4 was shown to be important for heart development^16,17^. VCAM1 is expressed in cardiomyocytes derived from human embryonic stem cells (hESCs) or human induced pluripotent stem cells (hiPSCs). These VCAM1-positive cells were found to express high levels of cardiac genes and display clear sarcomere structure, self-beating and action potentials similar to ventricular and pacemaker cells^18^.

In this report, we show that the DCM phenotype in the *VCAP1X2* mutant is attributed to sarcomere disorganization, decreased pAKT and pERK expression, and reduced gene expression for sarcomere modulators, sarcomeric proteins and calcium regulators. We conducted rescue experiments by injecting embryos with variant mRNAs for either *VCAP1X2* or *smyd1b* (a downstream effector), and probed the underlying signaling mechanisms by treating with PI3K and pMEK inhibitors or activators. Together, our results reveal two tyrosine residues located in the VCAP1X2 cytoplasmic domain are essential to regulate cardiac contractility and the proliferation of ventricular cardiomyocytes and epicardial cells through modulation of pAKT and pERK levels.

## Results

### VCAP1X2 is expressed in the heart and localized to myocardium plasma membrane

**Figure 1:**
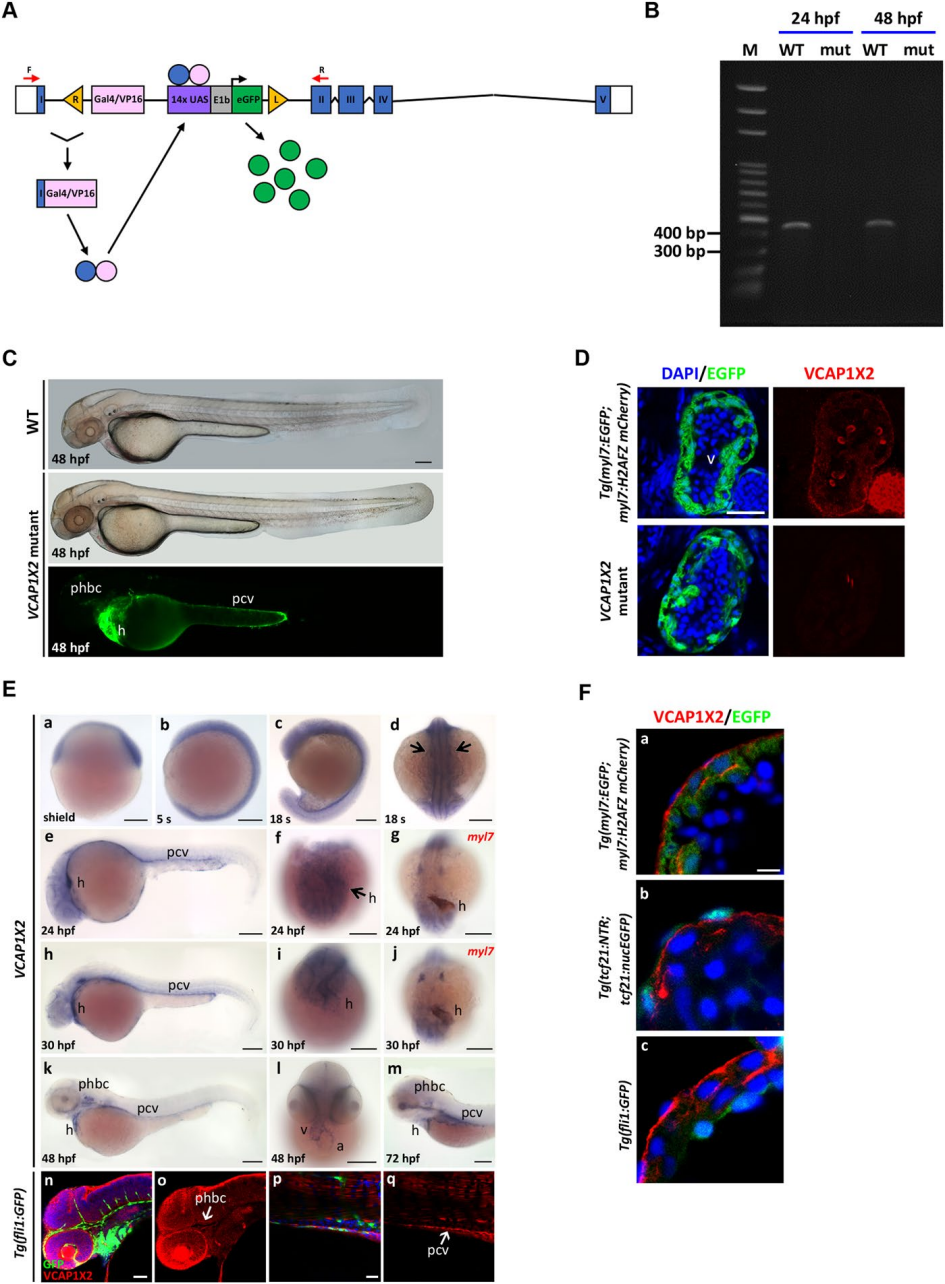
The gene trap transgenic zebrafish was characterized as a *VCAP1X2* mutant. (**A**) A diagram of the *VCAP1X2* gene trap mutant is shown. Blue boxes indicate exons I-V. White boxes indicate 5′ and 3′ UTRs. Red arrows indicate forward (F) and reverse (R) RT-PCR primers. Yellow triangles indicate 535-bp right Tol2 arm (R) or 517-bp left Tol2 arm (L). UAS, Gal4-responsive upstream activating sequence; E1b, adenoviral E1b minimal promoter element. (**B**) Splicing of exon 1 and exon 2 was disrupted in *VCAP1X2* mutant (mut) embryos at 24 and 48 hpf. (**C**) Normal morphology of *VCAP1X2* mutants and WT embryos at 48 hpf. EGFP expression was detected in the heart (h), posterior cardinal vein (pcv) and primordial hindbrain channel (phbc) of *VCAP1X2* mutant (n = 50 for 48 hpf). Scale bar, 25 μm. (**D**) Expression of EGFP and VCAP1X2 (red) is shown in heart ventricles of *Tg*(*myl7: EGFP; myl7: H2AFZ mcherry*) embryos at 96 hpf. No VCAP1X2 expression was detected in the ventricle of homozygous *VCAP1X2* mutant heart (n = 15 per condition, N = 3). Scale bar, 50 μm. (**E**) Expression of *VCAP1X2* at shield (a), 5 somite (s) (b), 18 s (c,d), 24 (e–g), 30 (h–j), 48 (k,l) and 72 (m) hpf stages. *VCAP1X2* was co-expressed with *myl7* in the myocardium of embryos at 24 and 30 hpf (g,j). Scale bars, 30 μm. Co-localization of VCAP1X2 and GFP was detected in phbc (n,o) and pcv (p,q) of *Tg(fli1:GFP)* embryos at 48 hpf (n = 10). Arrows indicate heart. a, atrium; h, heart; v, ventricle. Scale bars, 50 μm. (**F**) (a) VCAP1X2 (red) was expressed in the plasma membrane of ventricular myocardium cardiomyocytes labeled with EGFP in *Tg*(*myl7:EGFP; myl7:H2AFZ mCherry*) transgenic embryos at 96 hpf. (b) VCAP1X2 was not expressed in EGFP-labeled ventricular epicardium cells in *Tg*(*tcf21:NTR; tcf21:nucEGFP*) transgenic embryos at 96 hpf. (c) VCAP1X2 was also not expressed in the EGFP-labeled ventricular endocardium of *Tg*(*fli1:GFP*) transgenic embryos at 96 hpf (n = 15 per condition, N = 3). Nuclei were stained by DAPI. Scale bar, 5 μm.

To discover genes involved in cardiac development, we injected Tol2-Gal4-VP16; UAS: EGFP-Tol2 plasmid^19^ and produced a gene trap transgenic zebrafish with EGFP expression in the heart (Fig. 1A). The insertion was located in intron 1 of the *si:ch211-74m13.3* gene (ZFIN ID: ZDB-GENE-060503-219) on chromosome 3. Two splice variants (si:ch211-74m13.3-201, si:ch11-74m13.3-202) consisting of seven or five exons were produced. The variants encode vascular cell adhesion protein 1 isoform X1 (VCAP1X1) (XP_002661307.2, 551 amino acids) and vascular cell adhesion protein 1 isoform X2 (VCAP1X2) (XP_005164393.1, 360 amino acids). VCAP1X2 protein contains a signal peptide, one intercellular adhesion molecule (ICAM) domain, two immunoglobulin (Ig) superfamily domains, a transmembrane domain, and a cytosolic domain, while VCAP1X1 possesses two extra Ig domains (Supplementary Fig. S1A). Sequence comparison indicated that VCAP1X1 and VCAP1X2 are identical at amino acids 1-303 (Supplementary Fig. S1B). However, strong expression of *VCAP1X2*, but not *VCAP1X1*, was identified at 72 and 96 hours post fertilization (hpf) by RT-PCR using specific reverse primers for each isoform, indicating that only *VCAP1X2* was expressed during embryonic stages (Supplementary Fig. S1C,D).

RT-PCR analysis indicated splicing of exon 1 and exon 2 was blocked by the insertion, and only exon 1 of *VCAP1X2* was synthesized in *si:ch211-74m13.3* gene trap homozygous transgenic fish (Fig. 1B). Normal global embryonic morphology was observed in *si:ch211-74m13.3* gene trap homozygous embryos at 48 hpf and intense EGFP expression was detected in the heart, primordial hindbrain channel and posterior cardinal vein (Fig. 1C). We then generated a polyclonal antibody using amino acids number 31–308 as an antigen. With this antibody, we detected VCAP1X2 in the heart ventricle at 96 hpf in *Tg(myl7:EGFP; myl7:H2AFZ mCherry)* transgenic fish but not homozygous mutant embryos, confirming that this gene trap line is indeed a *VCAP1X2* mutant (Fig. 1D).

*VCAP1X2* was expressed ubiquitously at shield and somite stages (5 s and 18 s) (Fig. 1E,a–c). Expression of *VCAP1X2* was identified in the heart beginning from the 18 s stage and showed strong ventricular and weak atrial expression at 48 and 72 hpf (Fig. 1E,d,f,i,l,m). *VCAP1X2* was co-expressed with *myl7* in the myocardium at 24 and 30 hpf (Fig. 1E,g,j), and was also expressed in the primordial hindbrain channel and posterior cardinal vein during different developmental stages (Fig. 1E,e,h,k,m). VCAP1X2 immunofluoresence signal co-localized with GFP in the primordial hindbrain channel and posterior cardinal vein of *Tg(fli1:GFP)* embryos at 48 hpf (Fig. 1E,n–q).

To further clarify the localization of VCAP1X2 in the heart, we conducted immunofluorescence staining (Fig. 1F). VCAP1X2 was observed at the plasma membrane of EGFP-expressing ventricular myocardium cells in *Tg (myl7: EGFP; myl7: H2AFZ mcherry)* embryos at 96 hpf (Fig. 1F,a). No VCAP1X2 was detected in GFP-expressing outer epicardial cells of *Tg(tcf21:NTR; tcf21:nucEGFP)* embryos or in GFP-expressing inner endocardial cells of *Tg(fli1:GFP)* embryos (Fig. 1F,b,c). Thus, VCAP1X2 is a membrane protein that is exclusively expressed in the myocardium.

### *VCAP1X2* deficient embryonic heart displays a DCM phenotype

**Figure 2:**
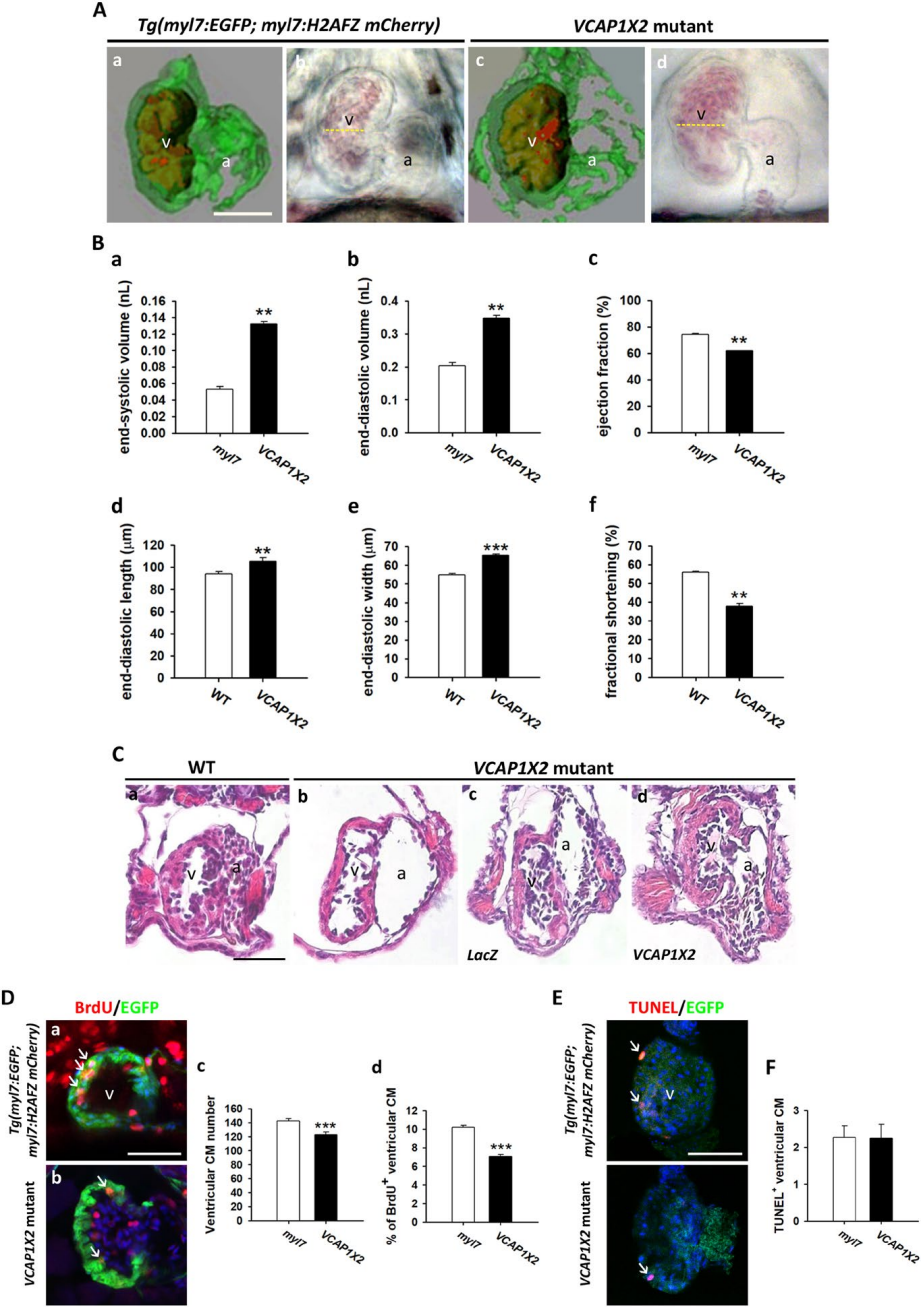
*VCAP1X2* mutant displayed cardiac dysfunction and impaired cardiomyocyte proliferation. (**A**) Pseudodynamic reconstructed 3D images and respective bright-field images are shown of end-diastolic phase beating hearts from *Tg(myl7:EGFP; myl7:H2AFZ mCherry)* (a,b) or *VCAP1X2* mutant (c,d) at 72 hpf. Yellow lines indicate the diameter of the ventricular chamber. Green fluorescence marks ventricular myocardium and red indicates ventricular chamber. Scale bar, 50 μm. (**B**) Parameters related to heart performance were measured from pseudodynamic reconstructed 3D images (a–c, n = 3, N = 2) or CCD images (d–f, n = 20, N = 3) for *Tg(myl7:EGFP; myl7:H2AFZ mCherry)* or WT and *VCAP1X2* mutant at 72 hpf. End-systolic volume (a) and end-diastolic volume (b) were significantly increased in *VCAP1X2* mutant compared with *Tg(myl7:EGFP; myl7:H2AFZ mCherry)* embryos. Ejection fraction (c) was lower in *VCAP1X2* mutant than in *Tg(myl7:EGFP; myl7:H2AFZ mCherry)* embryos. End-diastolic length (d) and width (e) were increased while fractional shortening (f) was decreased in *VCAP1X2* mutant compared to WT. Error bars represent standard error. Student’s *t*-test, **p < 0.01, ***p < 0.001. (**C**) Paraffin sectioning with hematoxylin and eosin staining revealed a thinner compact layer in the ventricle of *VCAP1X2* mutants (b) and *LacZ* mRNA-injected *VCAP1X2* mutants (c) compared to WT (a) or *VCAP1X2* mRNA-injected *VCAP1X2* mutants (d) (n = 10 per condition). a, atrium, v, ventricle. Scale bar, 50 μm. (**D**) Immunostaining of BrdU (red) in heart ventricles from *Tg(myl7:EGFP; myl7:H2AFZ mCherry)* (*myl7*) (a) or *VCAP1X2* mutants (b) at 72 hpf. Significantly decreased cardiomyocyte (CM) number (c) and percentage of BrdU^+^ CMs (d) in the ventricle was detected in *VCAP1X2* mutant compared to *Tg(myl7:EGFP; myl7:H2AFZ mCherry)* embryos (n = 20 per condition, N = 3). Student’s *t*-test, ***p < 0.001. Scale bar, 50 μm. Apoptotic cells were analyzed by TUNEL labeling (**E**) and similar low level of apoptotic cells (**F**) was detected in *VCAP1X2* mutant and *Tg(myl7:EGFP; myl7:H2AFZ mCherry)* embryos (n = 15, N = 3). Cardiomyocyte nuclei stained by anti-MEF2 antibody. Error bars represent standard error. Scale bar, 50 μm.

To characterize the role of VCAP1X2 in heart development, pseudodynamic 3D cardiac imaging was applied to zebrafish embryos, and cardiac function was evaluated at 72 hpf (Fig. 2A)^20^. Both end-systolic volume (ESV) and end-diastolic volume (EDV) were substantially increased, while ejection fraction (EF) was decreased in *VCAP1X2* mutant compared to *Tg (myl7: EGFP; myl7: H2AFZ mcherry)* controls (Fig. 2B,a–c). In parallel, we determined the end-diastolic length and width of *VCAP1X2* mutant hearts by recording a video of the beating heart. Increased end-diastolic length and width, with reduced fractional shortening (FS) were observed in *VCAP1X2* mutants relative to controls (Fig. 2B,d–f), further demonstrating that *VCAP1X2* mutant embryos have impaired cardiac function.

Histological analysis revealed a thinner ventricular compact layer (one-cell layer) in *VCAP1X2* mutant heart compared to WT (three-cell layer) embryos at 120 hpf, and injection of *VCAP1X2* full-length (FL) but not *LacZ* mRNA restored the thickness (Fig. 2C). Significantly reduced ventricular cardiomyocyte number and a decreased percentage of BrdU^+^ cardiomyocytes were detected in ventricles of *VCAP1X2* mutant compared to control embryos at 72 hpf (Fig. 2D,c,d), while similar low levels of apoptosis were detected in ventricles of *VCAP1X2* mutant and control hearts by TUNEL labeling at 72 hpf (Fig. 2E,F). Thus, the thinner ventricular compact layer with reduced cardiomyocyte number in *VCAP1X2* mutants may be due to decreased cell proliferation. Together, these results show that *VCAP1X2* deficient embryonic heart exhibits DCM phenotypes, including impaired cardiac function, enlarged ventricular chamber and thinner ventricular compact layer with decreased cardiomyocyte number.

### Two tyrosine residues within the VCAP1X2 cytoplasmic domain are required for heart contractility and cardiomyocyte proliferation

**Figure 3:**
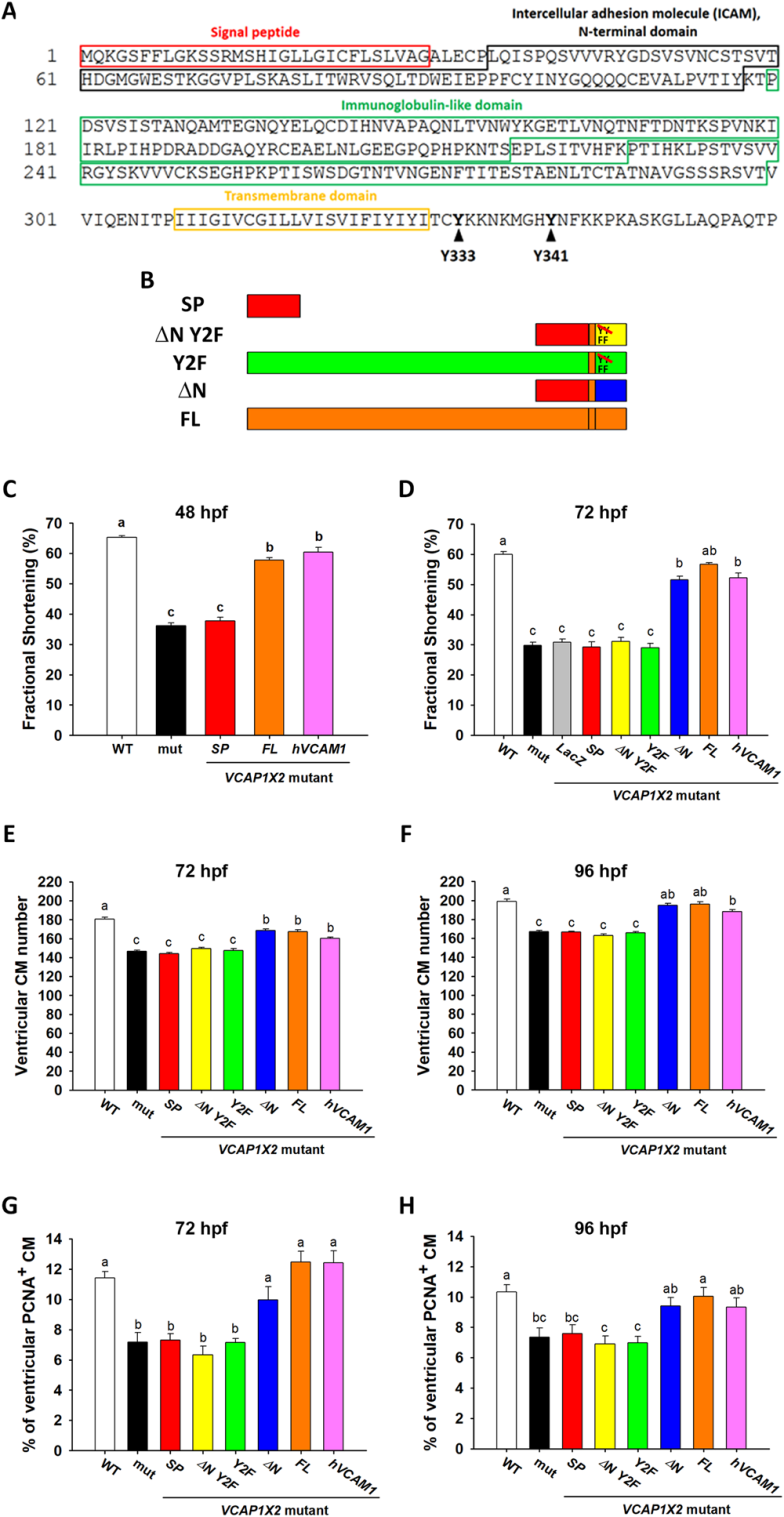
Full-length or *ΔN VCAP1X2* or human *VCAM1* mRNA rescue decreased fractional shortening and cardiomyocyte proliferation in *VCAP1X2* mutants. (**A**) Amino acid sequence and various domains of VCAP1X2 are shown. (**B**) A diagram shows various VCAP1X2 variants. (**C**) Ventricular fractional shortening (FS) was significantly lower in *VCAP1X2* mutant (mut) than in WT and the defect was rescued by injection with full-length (*FL*) *VCAP1X2* or human *VCAM1* mRNA (*hVCAM1*) but not with *SP VCAP1X2* mRNA at 48 hpf (n = 20 per condition, N = 3). (**D**) At 72 hpf, the FS defect was still observed in mutants and was rescued by injection with Δ*N*, *FL VCAP1X2*, or *hVCAM1* mRNA, but not with *LacZ*, *SP*, Δ*N Y2F* or *Y2F VCAP1X2* mRNA (n = 20 per condition, N = 3). (**E,F**) Ventricular cardiomyocyte (CM) number was significantly decreased in *VCAP1X2* mutant at 72 hpf (**E**) and 96 hpf (**F**) compared to WT. Injection of *ΔN* or *FL VCAP1X2 or hVCAM1* mRNA but not *SP*, *ΔN Y2F* or *Y2F VCAP1X2* mRNA could restore CM number in *VCAP1X2* mutant embryos (n = 20 per condition, N = 3). (**G,H**) A substantially reduced percentage of ventricular PCNA^+^ cardiomyocytes was detected in *VCAP1X2* mutant at 72 hpf (**G**) and 96 hpf (**H**) compared to WT. Injection of *ΔN* or *FL VCAP1X2* or *hVCAM1* mRNA but not *SP*, *ΔN Y2F* or *Y2F VCAP1X2* mRNA could restore the decreased percentage of ventricular PCNA^+^ cardiomyocytes in *VCAP1X2* mutant embryos at 72 hpf. However, at 96 hpf, only injection of *FL*, but not *ΔN* or *hVCAM1*, significantly restored percentage of ventricular PCNA^+^ cardiomyocytes in *VCAP1X2* mutants (n = 20 per condition, N = 3). Error bars indicate standard error. Quantitative data were analyzed by ANOVA with Bonferroni multiple comparisons. Treatments that are not statistically different (α = 0.05) from each other are labeled with the same letter.

Because phosphorylation of two cytosolic tyrosine residues in platelet endothelial cell adhesion molecule-1 (PECAM1) is known to activate extracellular signal-regulated kinase (ERK)^21^, we wondered if the two cytosolic tyrosine residues (Y333 and Y341) of VCAP1X2 may also play important functional roles (Fig. 3A). We generated five VCAP1X2 variants, including SP: only signal peptide, ΔN Y2F: deleted extracellular ICAM and Ig domains with two cytosolic tyrosines mutated to phenylalanines, Y2F: full-length VCAP1X2 with cytosolic tyrosine to phenylalanine mutations, ΔN: deleted extracellular ICAM and Ig domains with two cytosolic tyrosines intact, and FL: full-length VCAP1X2 (Fig. 3B). Initially, we compared heart contractility by measuring FS at 48 hpf in WT or *VCAP1X2* mutant embryos, or mutants injected with *SP* or *FL* mRNA. Injection of *FL*, but not *SP* mRNA, restored ventricular FS to levels comparable with WT (*p* < 0.001, Fig. 3C). Interestingly, injection of human vascular cell adhesion protein 1 isoform c precursor (*hVCAM1*) mRNA also rescued decreased FS in injected mutant embryos, despite the fact that VCAP1X2 only shares 27% amino acid sequence identity with human VCAM1 isoform c (Supplementary Fig. S2A). At 72 hpf, FS was restored in *VCAP1X2* mutants injected with *ΔN*, *FL* or *hVCAM1* mRNA, but not in those injected with *SP*, *ΔN Y2F*, *Y2F* or *LacZ* mRNA (*p* < 0.001, Fig. 3D). Furthermore, injection of *ΔN*, *FL* or *hVCAM1*, but not *SP*, *ΔN Y2F* or *Y2F* mRNA restored ventricular cardiomyocyte number (*p* < 0.001, Fig. 3E,F) at 72 and 96 hpf. Injection of *FL* or *hVCAM1* increased the percentage of ventricular PCNA^+^ cardiomyocytes in *VCAP1X2* mutants more robustly (*p* < 0.001) than *ΔN* (*p* < 0.05) at 72 hpf (Fig. 3G). However, at 96 hpf, only injection of *FL*, but not *ΔN* or *hVCAM1*, significantly restored percentage of ventricular PCNA^+^ cardiomyocytes in *VCAP1X2* mutants (*p* < 0.001, Fig. 3H).

We then designed a splicing morpholino (spMO) that targets the acceptor site of intron 1 and monitored the inhibition of splicing between exon 1 and exon 2 of *VCAP1X2* by RT-PCR (Supplementary Fig. S3A,B). VCAP1X2 immunofluorescence was detected in heart ventricles at 96 hpf in *Tg(myl7:EGFP; myl7:H2AFZ mCherry)* transgenic fish and 5 mm spMO-injected control embryos but not spMO-injected morphants (Supplementary Fig. S3C). A thinner ventricular compact layer was detected in *VCAP1X2* morphant heart, which was rescued by co-injection of *VCAP1X2* but not *LacZ* mRNA (Supplementary Fig. S3D). Co-injection of *ΔN* or *FL*, but not of *SP*, *ΔN Y2F* or *Y2F* mRNA also completely restored reduced ventricular FS and ventricular cardiomyocyte number (*p* < 0.001, Supplementary Fig. S3E,F). Together, these results demonstrate that the two cytosolic tyrosine residues of VCAP1X2 are essential for heart contractility, compact layer thickness and cardiomyocyte proliferation in the ventricle of embryonic heart.

### VCAP1X2 cytosolic tyrosine residues are essential for maintaining pAKT and pERK levels

Cardiomyocyte proliferation is regulated by several signaling pathways, including neuregulin-1/ErbB2^22^ and insulin like growth factor 2 signaling^23^. PI3K-AKT and MAPK-ERK1/2 transduce upstream signals to activate genes involved in different biological processes, including cell proliferation. Since ventricular cardiomyocyte proliferation was reduced in *VCAP1X2* mutants (Fig. 2), we investigated whether pAKT and pERK levels were also affected in the ventricle of *VCAP1X2* mutant embryonic hearts.

**Figure 4:**
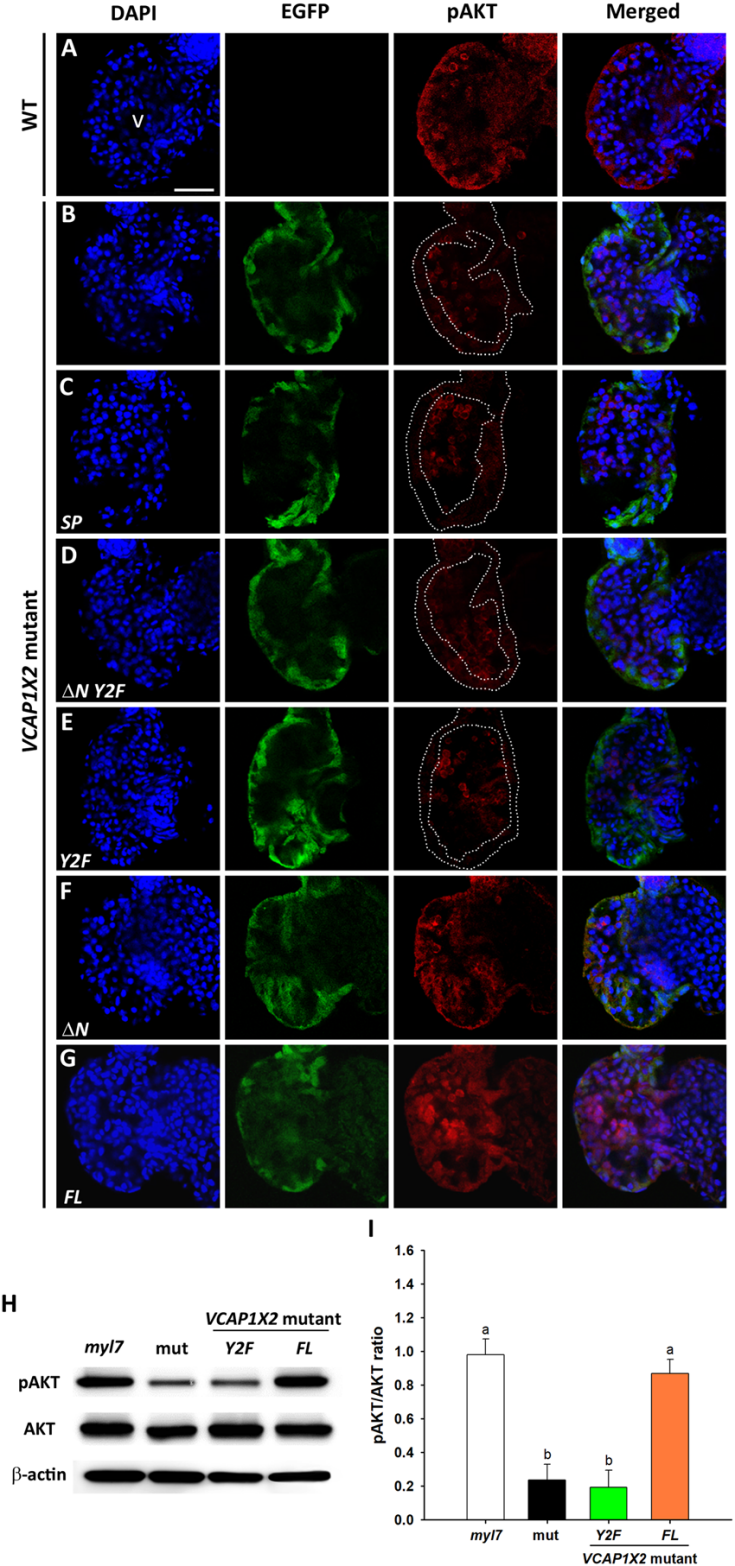
Maintenance of pAKT required expression of two tyrosines in the cytoplasmic domain of VCAP1X2. Immunofluorescent staining revealed decreased pAKT expression levels in *VCAP1X2* mutants (**B**) compared to WT (**A**) at 96 hpf. pAKT expression levels in *VCAP1X2* mutants could be restored by injection with *ΔN* (**F**) or *FL* (**G**) *VCAP1X2* mRNA but not with *SP* (**C**), *ΔN Y2F* (**D**) or *Y2F* (**E**) *VCAP1X2* mRNA (n = 15 per condition, N = 3). Nuclei were stained with DAPI. Scale bar, 30 μm. v, ventricle. Dashed lines illustrate the ventricular myocardium. Erythrocytes in the ventricular chamber show non-specific staining from secondary antibody. (**H**) Levels of pAKT or total AKT in embryonic hearts isolated from *Tg*(*myl7:EGFP; myl7:H2AFZ mCherry*) (*myl7*), *VCAP1X2* mutant (mut) or *VCAP1X2* mutant injected with *Y2F* or *FL VCAP1X2* mRNA were measured by Western blot with β-actin as loading control. Cropped representative blots for each protein are shown. Uncropped blots are shown in Supplementary Fig. S4. All blotting was performed using the same experimental conditions. (**I**) Quantification of pAKT/AKT expression ratio among different treatments (N = 3). Error bars indicate standard error. Quantitative data were analyzed by ANOVA with Bonferroni multiple comparisons. Treatments that are not statistically different (α = 0.05) from each other are labeled with the same letter.

**Figure 5:**
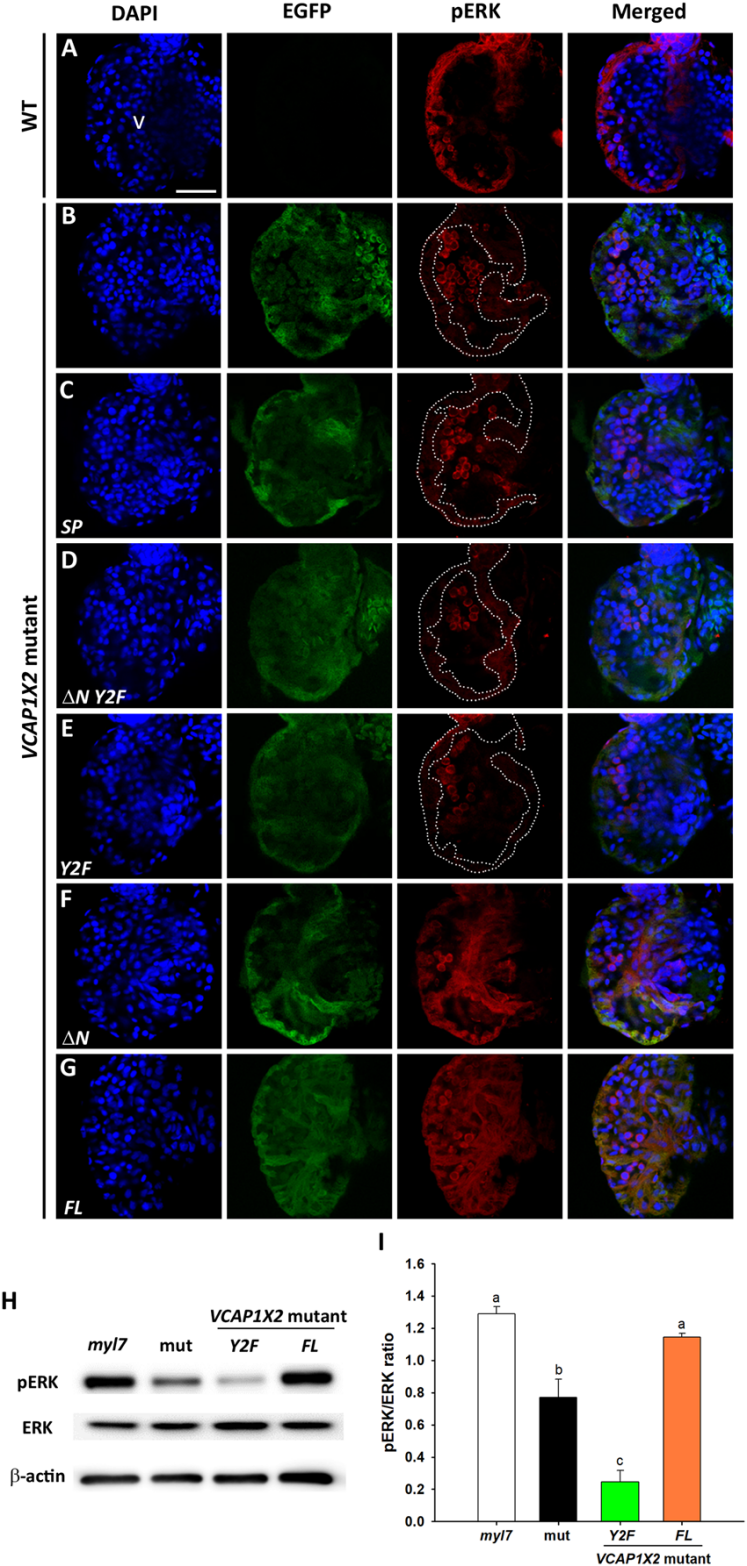
Maintenance of pERK required expression of two tyrosines in the cytoplasmic domain of VCAP1X2. Immunofluorescent staining revealed reduced pERK expression levels in *VCAP1X2* mutant embryos (**B**) compared to WT (**A**) at 96 hpf. Reduced pERK expression levels in heart ventricles of *VCAP1X2* mutants could be restored by injection with *ΔN* (**F**) or *FL* (**G**) *VCAP1X2* mRNA but not with *SP* (**C**), *ΔN Y2F* (**D**) *or Y2F* (**E**) *VCAP1X2* mRNA (n = 15 per condition, N = 3). Dashed lines illustrate the ventricular myocardium. Erythrocytes in the ventricular chamber show non-specific staining from secondary antibody. Nuclei were stained with DAPI. Scale bar, 30 μm. v, ventricle. (**H**) Levels of pERK or total ERK in embryonic hearts isolated from *Tg*(*myl7:EGFP; myl7:H2AFZ mCherry*) (*myl7*), *VCAP1X2* mutant (mut) or *VCAP1X2* mutant injected with *Y2F* or *FL VCAP1X2* mRNA were measured by Western blot analyses with β-actin as loading control. Cropped representative blots for each protein are shown. Uncropped blots are shown in Supplementary Fig. S4. All blotting was performed using the same experimental conditions. (**I**) Quantification of pERK/ERK expression ratio among different treatments (N = 3). Error bars indicate standard error. Quantitative data were analyzed by ANOVA with Bonferroni multiple comparisons. Treatments that are not statistically different (α = 0.05) from each other are labeled with the same letter.

Both pAKT and pERK were diminished in *VCAP1X2* mutant heart compared to WT at 96 hpf (Figs 4A,B and 5A,B). Injection of *ΔN* or *FL* mRNA, but not *SP*, *ΔN Y2F* or *Y2F* mRNA restored ventricular pAKT and pERK levels (Figs 4C–G and 5C–G). Western blots further revealed reductions in pAKT (4.2-fold) and pERK (1.8-fold) levels in *VCAP1X2* mutant heart (Figs 4H,I, 5H,I, Supplementary Fig. S4). Injection of *FL*, but not *Y2F* mRNA restored pAKT and pERK to levels that were comparable to *Tg (myl7: EGFP; myl7: H2AFZ mcherry)* embryonic hearts at 96 hpf (*p* < 0.05, Figs 4I, 5I). Notably, *Y2F* mRNA injection further reduced ventricular pERK level in *VCAP1X2* mutant hearts (*p* < 0.01, Fig. 5I).

*VCAP1X2* variant mRNAs were overexpressed in *Tg (myl7: EGFP; myl7: H2AFZ mcherry)* transgenic embryos to investigate the effects on cardiomyocyte number, and pAKT and pERK levels. Reductions in pAKT, pERK and cardiomyocyte number were identified in ventricles of transgenic embryos overexpressing *ΔN Y2F* or *Y2F*, but not *ΔN* or *FL* mRNA at 96 hpf (Supplementary Figs S5C–F, S6C–F, S7). Together, these results indicate that the two cytosolic tyrosine residues of VCAP1X2 are essential for maintaining proper ventricular pAKT and pERK levels as well as cardiomyocyte number in embryonic hearts.

### *VCAP1X2* deficiency and inhibition of pAKT or pERK induced sarcomere disorganization

**Figure 6:**
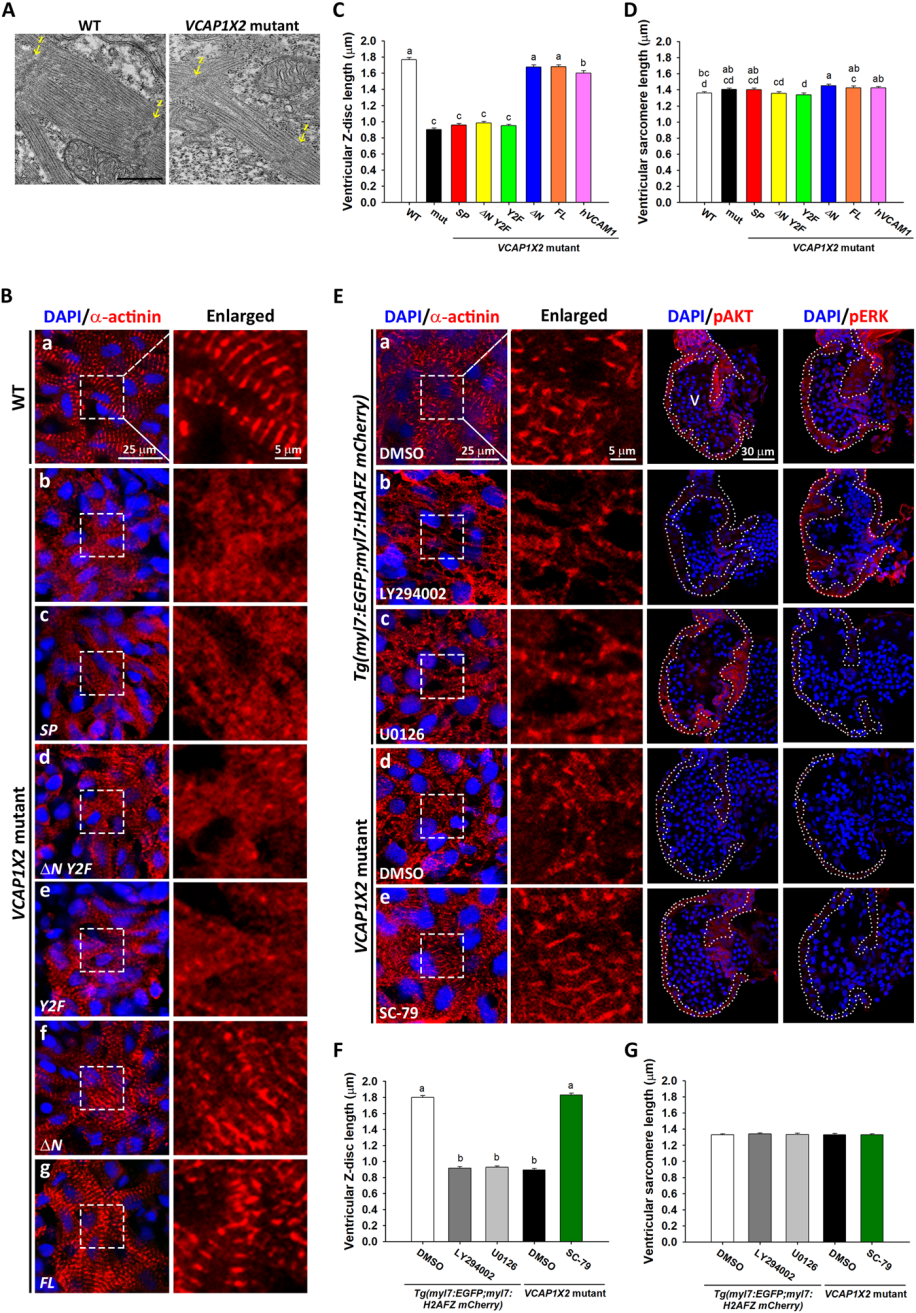
Disorganized sarcomere structure was observed in *VCAP1X2* mutant hearts and rescued by *VCAP1X2* variant mRNA, *hVCAM1* mRNA or a PI3K activator. (**A**) Transmission electron microscopy shows sparse myofilaments and indistinct Z-discs in ventricular sarcomere of *VCAP1X2* mutants at 96 hpf (n = 3 per condition). Arrows indicate Z-discs (Z). Scale bar, 500 nm. (**B**) Striated Z-discs were detected by α-actinin immunofluorescence in WT heart (a), while a dotted pattern was observed in *VCAP1X2* mutants (b) at 96 hpf. *ΔN* (f) or *FL* (g), but not *SP* (c), *ΔN Y2F* (d) or *Y2F* (e), restored striated Z-discs (n = 16 per condition, N = 3). Z-disc length and sarcomere length were measured from immunofluorescence images. Z-disc length (**C**) was reduced in *VCAP1X2* mutants and *SP*, *ΔN Y2F* or *Y2F*-injected mutants but not those injected with *ΔN*, *FL*, or *hVCAM1* mRNA at 96 hpf (n = 160 per condition, N = 3). (**D**) Slight differences (maximum 8%) were found in sarcomere length, indicating that ventricular sarcomere length was not robustly affected by any treatment at 96 hpf. Data in (**C** and **D**) were analyzed by ANOVA with Bonferroni multiple comparisons. Treatments that are not statistically different (α = 0.05) from each other are labeled with the same letter. (**E**) α-actinin immunofluorescence on sarcomeres of DMSO-treated *Tg*(*myl7:EGFP; myl7:H2AFZ mCherry*) embryos (a) or PI3K activator (SC-79)-treated *VCAP1X2* mutant heart (e) showed striations, while *Tg*(*myl7:EGFP; myl7:H2AFZ mCherry*) embryos treated with inhibitors of PI3K (LY294002) (b) or pMEK (U0126) (c), or DMSO-treated *VCAP1X2* mutant hearts (d) showed a dotted pattern. Ventricular myocardium is outlined in right-most panels. Erythrocytes show non-specific staining from secondary antibody. (**F**) Z-disc length was measured in *Tg*(*myl7:EGFP; myl7:H2AFZ mCherry*) embryos and *VCAP1X2* mutants treated with PI3K or pMEK inhibitor. Data were analyzed by ANOVA with Bonferroni multiple comparisons (α = 0.05). The difference between groups a and b was significant (*p* < 0.001). (**G**) Similar ventricular sarcomere length was detected in all groups (n = 200 per condition, N = 3). ANOVA indicated no significant difference among treatments (*p* = 0.96). Error bars indicate standard error.

Since heart contractility was decreased in *VCAP1X2* mutants (Fig. 2), we investigated whether sarcomere assembly was affected in the ventricle. Sarcomere organization was examined by transmission electron microscopy, which revealed intact and well-organized structures with clear Z-disc boundaries in WT heart at 96 hpf. By contrast, sparse and irregular myofibrils bordered by blurred Z-discs were observed in *VCAP1X2* mutant heart (Fig. 6A). Blurred Z-discs were also detected by α-actinin immunofluorescence in *VCAP1X2* mutant heart compared to WT at 96 hpf (Fig. 6B,a,b). Injection of *ΔN* or *FL* mRNA, but not *SP*, *ΔN Y2F* or *Y2F* mRNA rescued the blurred Z-discs (Fig. 6B,c–g). We further measured the resting Z-disc length and sarcomere length in the embryonic hearts. Compared to WT, *VCAP1X2* mutant heart ventricles exhibited highly significantly reduced Z-disc length (*p* < 0.001, Fig. 6C). Again, injection of *ΔN*, *FL* or *hVCAM1* mRNA, but not *SP*, *ΔN Y2F* or *Y2F* mRNA restored the defect (*p* < 0.001, Fig. 6C). Similar sarcomere lengths were measured in all groups (Fig. 6D). Therefore, we conclude that two cytosolic tyrosine residues of VCAP1X2 are essential in regulating sarcomere organization.

Since sarcomere disorganization and decreased pAKT and pERK were detected in the ventricles of *VCAP1X2* mutant hearts (Figs 4–6), we wondered whether inhibition of pAKT or pERK expression in WT embryos would affect sarcomere organization. We treated WT embryos with an inhibitor of PI3K (LY294002) or pMEK(U0126) at 63 hpf and fixed embryos for α-actinin immunofluorescence at 72 hpf. Blurred shortened Z-discs were detected in heart ventricles of *Tg (myl7: EGFP; myl7: H2AFZ mcherry)* embryos treated with PI3K or pMEK inhibitor compared to DMSO-treated embryos (Fig. 6E,a–c,F,G), but sarcomere length was similar. We also treated *VCAP1X2* mutant embryos with pAKT activator (SC-79) for a similar period prior to α-actinin immunostaining. Complete restoration of Z-disc alignment and length were detected in heart ventricles of SC-79-treated *VCAP1X2* mutants compared to DMSO-treated controls (Fig. 6E,d,e,F,G). Together, these results demonstrate that two cytosolic tyrosine residues of VCAP1X2 and proper pAKT and pERK levels are essential for sarcomere organization in the heart ventricle.

### Two cytosolic tyrosine residues of VCAP1X2 are essential for epicardium formation and epicardial cell proliferation

A prior study revealed *VCAM1*-deficient mouse hearts exhibited thinned compact layer, reduction of intraventricular septum and epicardium formation defects^16^. Because *VCAP1X2* mutant embryonic heart showed similar defects of thinner ventricular compact layer and reduced cardiomyocyte number (an effect that could be rescued by *hVCAM1* mRNA; Figs 2, 3), we next investigated whether epicardium formation was also affected in *VCAP1X2* mutants. Indeed, ventricular epicardium formation was affected in *VCAP1X2* mutants, as evidenced by *tcf21* RNA staining at 96 hpf (Supplementary Fig. S8A). To further investigate the effect of *VCAP1X2* deficiency on epicardium formation, we knocked down *VCAP1X2* in *Tg(tcf21:nucEGFP)* transgenic embryos and labeled proliferative epicardial cells with PCNA antibody at 96 hpf (Supplementary Fig. S8B). A reduced number of ventricular Tcf21^+^ epicardial cells was observed in *VCAP1X2* morphants and co-injection of *ΔN* or *FL*, but not *SP*, *ΔN Y2F* or *Y2F* mRNA, completely restored Tcf21^+^ epicardial cell number compared to un-injected or 5 mm spMO-injected transgenic embryos (*p* < 0.001, Supplementary Fig. S8C). Similarly, a reduction in the percentage of ventricular Tcf21^+^/PCNA^+^ cells was completely rescued by co-injection of Δ*N* or *FL*, but not *SP*, *ΔN Y2F* or *Y2F* mRNAs. (*p* < 0.001, Supplementary Fig. S8D). Thus, the two cytosolic tyrosine residues of VCAP1X2 are also required for proper epicardial cell proliferation and epicardium formation.

### *VCAP1X2* deficiency leads to reduced gene expression for proteins involved in sarcomere assembly and calcium homoeostasis

**Figure 7:**
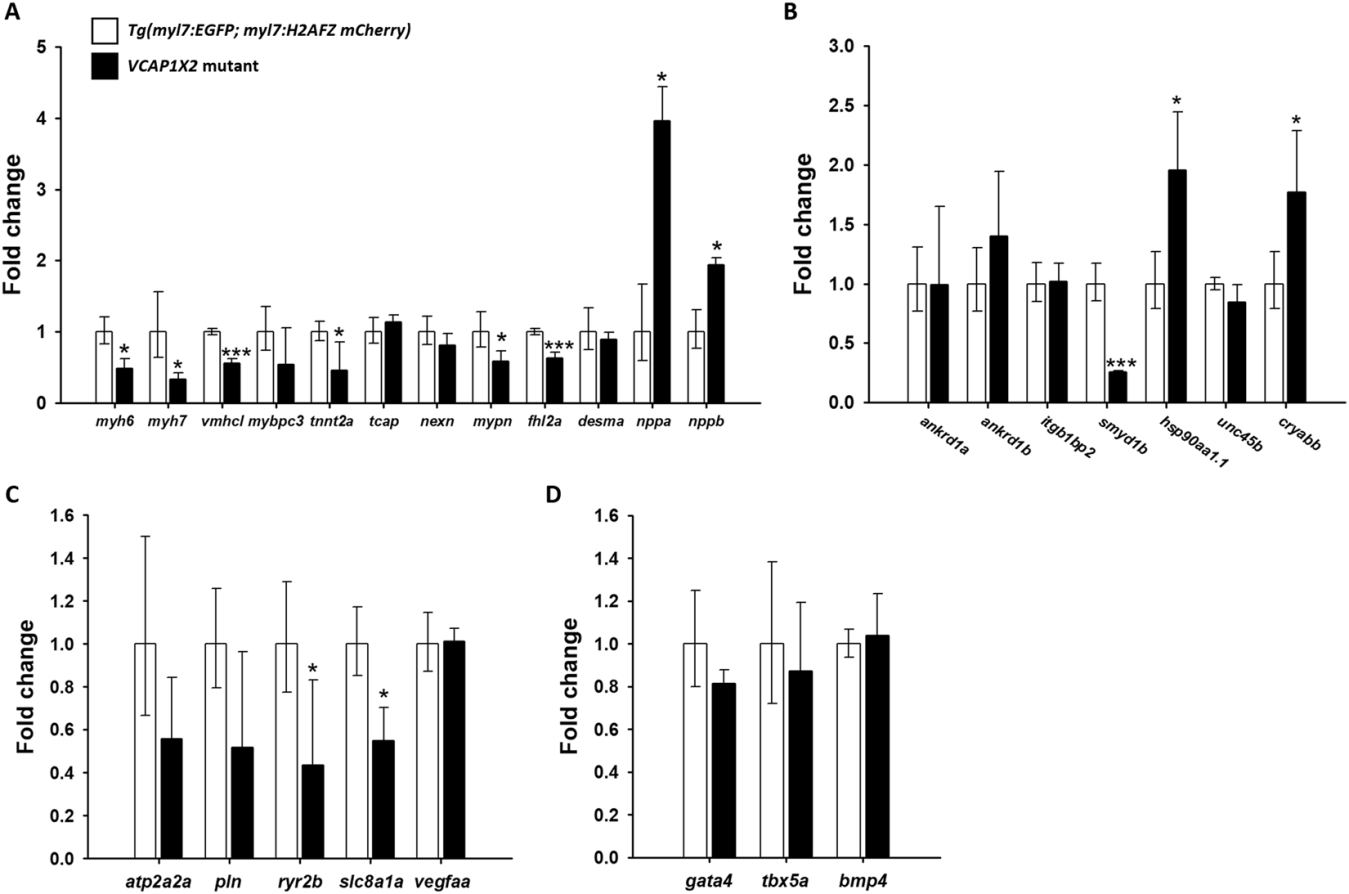
Real-time reverse-transcription PCR revealed altered expression of genes related to sarcomere assembly and heart contractility in embryonic hearts from *Tg (myl7: EGFP; myl7: H2AFZ mcherry)* and *VCAP1X2* mutant at 96 hpf. (**A**) Dilated cardiomyopathy-related genes encoding sarcomeric proteins, including *myh6*, *myh7*, *vmhcl*, *tnnt2a*, *mypn* and *fhl2a*, were significantly decreased, while expression of two natriuretic peptides (*nppa* and *nppb*) were substantially increased. (**B**) *smyd1b* was significantly reduced in *VCAP1X2* mutant accompanied by an induction of expression of *hsp90aa1.1* and *cryabb*. (**C**) Ca^2+^ handling regulators (*ryr2b* and *slc8a1a*) were significantly downregulated in *VCAP1X2* mutants. (**D**) Genes related to epicardium formation were not significantly altered between WT and *VCAP1X2* mutants. Error bars indicate standard deviation. Student’s *t*-test, *p < 0.05. ***p < 0.001.

To further investigate the mechanism by which VCAP1X2 regulates heart contractility and epicardium formation, we compared gene expression levels of proteins involved in sarcomere assembly, calcium homeostasis, and epicardium formation. Embryonic hearts of *VCAP1X2* mutant and WT zebrafish were evaluated by RT-qPCR at 96 hpf (Fig. 7). Since *VCAP1X2* mutant embryonic heart exhibited a DCM phenotype, we selected several zebrafish orthologues of known human DCM-associated genes for comparison^24^ (Fig. 7A). Expression of genes encoding sarcomere proteins, such as myosin heavy chain 6 (*myh6)*, myosin heavy chain 7 (*myh7*), ventricle myosin heavy chain like (*vmhcl*), troponin T (*tnnt2a*), myopalladin (*mypn*) and four and a half LIM domains 2a (*fhl2a*), was significantly downregulated in *VCAP1X2* mutants compared to WT. Expression levels of two natriuretic peptides (*nppa*, *nppb*) were substantially increased in *VCAP1X2* mutant heart, similar to other DCM animal models^25^. We then compared expression levels of the sarcomere modulator, *smyd1b*^6^, stress response regulators [integrin subunit beta1 binding protein 2 (*itgb1bp2*)^26^ and ankyrin repeat domain 1 (*ankrd1*)^27^] and chaperones (*hsp90aa1.1*, *unc45b*, αB-crystallin/*cryabb*)^28,29^ in *VCAP1X2* mutant and WT hearts (Fig. 7B). Significant downregulation of *smyd1b*, and upregulation of *hsp90aa1.1* and *cryabb* were detected in *VCAP1X2* mutants.

Because the contraction and relaxation of cardiac muscle is regulated by cytosolic Ca^2+^ concentration^30^, we investigated whether expression of calcium regulators was affected in *VCAP1X2* mutant hearts (Fig. 7C). Diminished expression of sarcoplasmic reticulum (SR) ryanodine receptor 2b (*ryr2b*) and sarcolemma solute carrier family 8, member 1a (*slc8a1a*) were detected in *VCAP1X2* mutants. We also compared gene expression for proteins that are essential for epicardium formation, such as GATA4, Tbx5 and BMP4^31,32^. A trend toward downregulation of *gata4* and *tbx5a* was observed in mutants, with no alteration of *bmp4* expression (Fig. 7D). Together, these finding suggest that VCAP1X2 regulates cardiac contractility by modulating expression of *smyd1b*, *mypn* or *fhl2a* for proper sarcomere assembly and Ca^2+^ regulatory proteins (*ryr2b*, *slc8a1a*) for calcium homeostasis.

### s*myd1b* mRNA injection restored cardiac dysfunction, sarcomere organization and cardiomyocyte proliferation in *VCAP1X2* mutant embryonic heart

**Figure 8:**
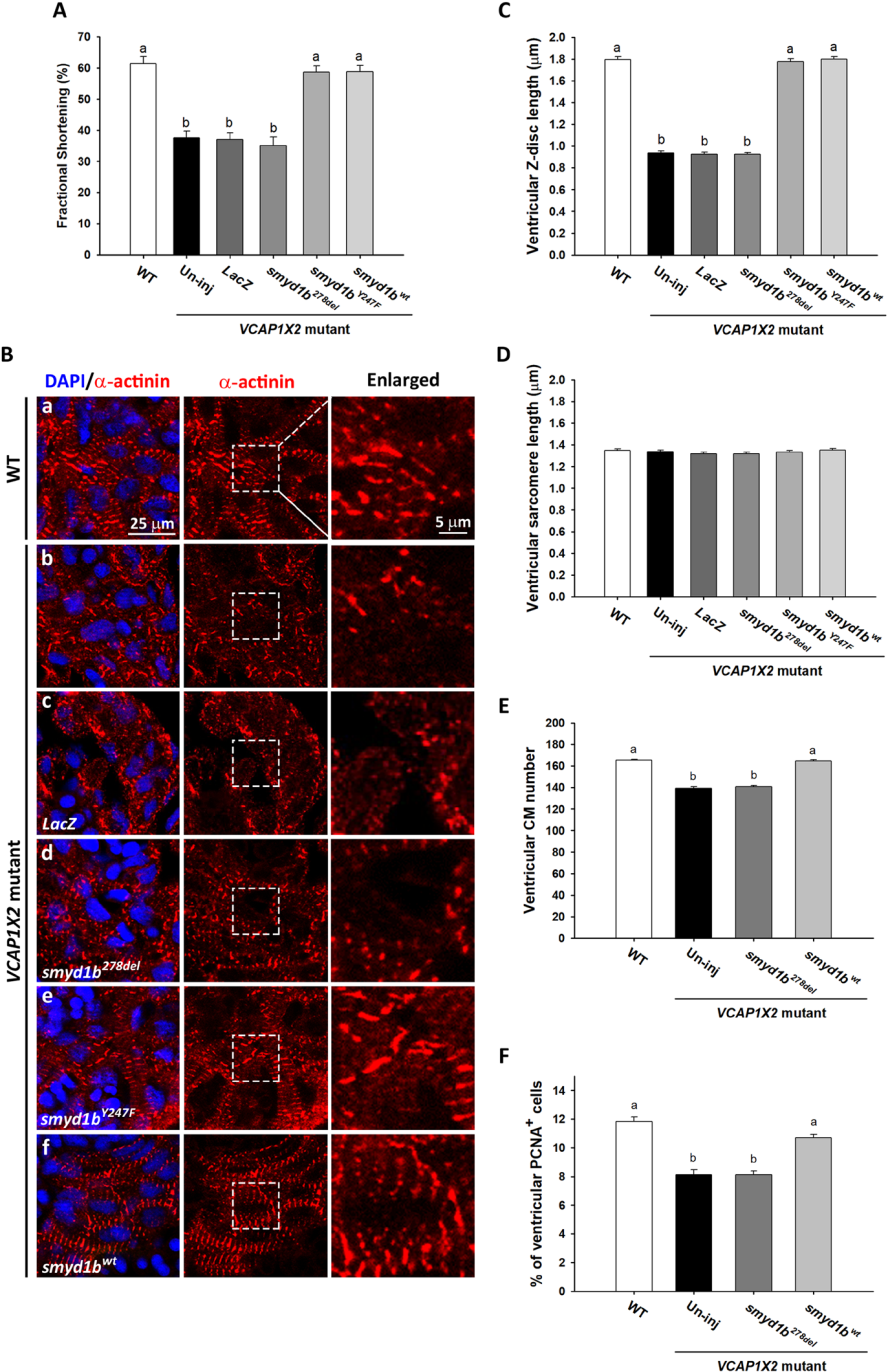
Injection of *smyd1b*^*wt*^ but not myosin-interaction-site-absent s*myd1b*^*278del*^ mRNA restored fractional shortening, sarcomere organization and cardiomyocyte proliferation. Injection of *smyd1b*^*wt*^ or HMT-deficient *smyd1b*^*Y247F*^ mRNA but not s*myd1b*^*278del*^ or *LacZ* mRNA restored fractional shortening. (**A**) (n = 24 per condition, N = 4) and Z-disc length (**C**) (n = 200 per condition, N = 4) at 96 hpf. Data were analyzed by ANOVA with Bonferroni multiple comparisons (α = 0.05). The difference between groups a and b was significant (*p* < 0.001). Similar ventricular sarcomere lengths (**D**) were detected in all groups (n = 200 per condition, N = 4). ANOVA indicated no significant difference among treatments (*p* = 0.49). (**B**) Striated Z-discs were detected by α-actinin immunofluorescence on sarcomeres in the ventricle of WT (a), *smyd1b*^*Y247F*^ (e) or *smyd1b*^*wt*^ (f) mRNA-injected *VCAP1X2* mutant hearts while a dotted pattern on Z-discs was observed in ventricles of *VCAP1X2* mutant (b), *LacZ* (c) or s*myd1b*^*278del*^ mRNA (d)-injected mutant hearts. Enlarged areas are indicated by white dashed rectangles. Nuclei were stained by DAPI. Significantly reduced cardiomyocyte number (**E**) or percentage of PCNA^+^ cardiomyocytes (**F**) was detected in ventricles of s*myd1b*^*278del*^ mRNA-injected or un-injected (Un-inj) *VCAP1X2* mutant hearts compared to *smyd1b*^*wt*^ mRNA-injected or WT embryos at 96 hpf (n = 15, N = 3). Data were analyzed by ANOVA with Bonferroni multiple comparisons. Treatments that are not statistically different (α = 0.05) from each other are labeled with the same letter. Error bars indicate standard error.

We focused on downregulation of *smyd1b* as a primary effector in *VCAP1X2* mutants, because Smyd1-myosin interaction is essential for thick filament assembly, and zebrafish *fla/smyd1* mutants exhibit disrupted sarcomere assembly in both heart and skeletal muscle^6^. In a rescue experiment, injection of *smyd1b*^*wt*^ or HMT-deficient *smyd1b*^*Y247F*^ mRNA, but not myosin-interaction-site-absent s*myd1b*^*278del*^ or *LacZ* mRNA, restored FS, blurred Z-discs and Z-disc length in heart ventricles of *VCAP1X2* mutants at 96 hpf (Fig. 8A–D). Furthermore, the reduced cardiomyocyte number and decreased percentage of PCNA^+^ cardiomyocytes were also restored at 96 hpf in mutant embryos injected with *smyd1b*^*wt*^ but not with s*myd1b*^*278del*^ mRNA (*p* < 0.001, Fig. 8E,F). Together, these finding suggest that VCAP1X2 regulates cardiac contractility and cardiomyocyte proliferation by modulating expression of *smyd1b*, a myosin-interacting protein that is required for proper sarcomere assembly.

## Discussion

The costamere-Z-disc interaction is important for transmission of mechanical force and molecular signals between the sarcomere, sarcolemma and extracellular matrix. Identifying proteins that connect the sarcolemma to sarcomeres can further our understanding of mechanical and signal transduction in cardiac muscle. Here, we identified a novel cell adhesion molecule, VCAP1X2, which is expressed in the sarcolemma and functions as an important signal transducer from the sarcolemma to the sarcomere and nucleus in cardiac muscle. This protein maintains cardiac contractility as well as proliferation of cardiomyocytes and epicardial cells in zebrafish embryonic heart ventricle.

*VCAP1X2* deficient embryonic hearts exhibited DCM phenotypes, including enlarged ventricular chamber, thinner ventricular compact layer with decreased cardiomyocyte number, and impaired cardiac function. Sparse and irregular myofilaments, bordered by blurred Z-discs with decreased length, were observed in *VCAP1X2* mutant heart and may be attributed to decreased expression of a sarcomere modulator (*smyd1b*), proteins associated with Z-discs and I-bands (*mypn*, *fhl2a*) and calcium regulators (*ryr2b*, *slc8a1a*). Smyd1b is expressed in cardiomyocytes, where it localizes to the M-line of the sarcomere and interacts with myosin. Disrupted myofibrillogenesis was previously reported in cardiomyocytes of zebrafish *fla* mutants with defective *smyd1b*. Knockdown of *smyd1b* induced upregulation of two chaperones (*hsp90*α*1* and *unc45b*) and resulted in cardiac sarcomere disorganization^33^. Although *unc45b* expression was not increased in *VCAP1X2* mutant heart, *cryabb*, which binds to titin in the I-band and protects it from stress^28^, was significantly induced. These upregulated chaperones may be responding to misfolded thick filament myosin during myofibrillogenesis. Furthermore, injection of *smyd1b*^*wt*^ but not myosin-interaction-site-absent s*myd1b*^*278del*^ mRNA restored FS, Z-disc alignment and Z-disc length in *VCAP1X2* mutants, suggesting that Smyd1b-mediated thick filament assembly is regulated by VCAP1X2.

In addition to Smyd1b, Mypn binds α-actinin at the Z-disc and interacts with ANKRD1 in the I-band to maintain sarcomere integrity^34^. While ANKRD1 is a transcriptional regulator, it also interacts with titin, where it serves as a scaffold to facilitate ERK1/2 phosphorylation of GATA4. Upon GATA4 phosphorylation, the ANKRD1/ERK/GATA4 complex translocates to the nucleus to regulate hypertrophic gene expression^27^. GATA4 is known to regulate cardiac muscle-specific gene (*α-MHC*, *β-MHC* and *MLC*) expression^35^ and promoted cardiomyocyte proliferation by modulating cardiomyocyte cell cycle activity^36^. In *VCAP1X2* mutant embryonic heart, decreased *smyd1b* expression affected thick filament assembly and sarcomere organization and downregulation of *mypn* may disrupt the interaction between Mypn and Ankrd1a to affect Ankrd1a/ERK/GATA4 complex formation and decrease ERK phosphorylation. Reduced GATA4 phosphorylation would affect its transcriptional activity, resulting in decreased expression of thick filament genes (*myh6*, *myh7*, *vmhcl*) and reduced expression of cell cycle genes such as *cyclin D2*, *cyclin A2* and *CDK4*^37^. As a result, reduced cardiomyocyte proliferation was detected in ventricles of *VCAP1X2* mutant hearts, which was restored by injection of *smyd1b*^*wt*^ but not s*myd1b*^*278del*^ mRNA. In addition, GATA4 was reported to be an essential factor for the production of proepicardium in mice, and decreased GATA4 transcriptional activity may also affect the epicardium formation in *VCAP1X2* mutant zebrafish^31^.

Besides the effects on cardiac muscle or cell cycle gene expression by reduced ERK-mediated GATA4 transcriptional activity, PI3K-AKT signaling was also shown to promote expression of genes involved in cardiac structure and Z-disc signaling^38^. Experiments using caPI3K and dnPI3K in transgenic mice indicated that genes encoding several cardiac muscle-related proteins, including Melusin (*Itgb1bp2*), were differentially expressed. Melusin was shown to associate with the cytosolic domain of integrin β1 at the costamere and interact with the p85 regulatory subunit of PI3K. Thinner myofibrils and reduced Z-disc alignment were identified in dnPI3K transgenic heart, and PI3K (p110α) inhibitors prevented the formation of mature myofibers in response to IGF1 stimulation. Therefore, upon IGF1 stimulation, PI3K interacts with insulin receptor substrate 1 and Melusin to activate downstream AKT kinase and regulate expression of several cardiac muscle-associated proteins. Consistent with these studies in mice, we also detected blurred Z-discs with reduced length in heart ventricles of zebrafish embryos treated with inhibitor of PI3K or pMEK. Furthermore, such defects in ventricles of *VCAP1X2* mutant hearts could be completely restored by treatment with PI3K activator. The PI3K-AKT pathway interacts with Wnt/β-catenin or Hippo-Yap signaling to regulate cardiomyocyte proliferation^36^. PI3K-AKT promoted β-catenin nuclear translocation by inhibiting GSK3β phosphorylation and induced Yap nuclear translocation by dissociation of the Mst-Sav-LATS1/2 complex and the inactivation of LATS1/2 via recruiting PDK1 to the plasma membrane^39^. β-catenin and Yap then interact with cofactors to activate expression of cell cycle genes.

Mammalian VCAM1 isoforms contain a highly conserved 19-amino-acid cytoplasmic domain that is not found in VCAP1X2 or zebrafish VCAM1 (Supplementary Fig. S2B). Within the 19-amino-acid sequence, S728 and Y729 are necessary for mouse VCAM1 activation of calcium flux, while S730 and S737 are required for VCAM1 activation of Rac1 during leukocyte transendothelial migration^40^. Similarly, VCAP1X2 possesses a short 30-amino-acid cytoplasmic domain with two tyrosine residues. We demonstrated that these two cytosolic tyrosine residues are essential for VCAP1X2 function. Although the VCAP1X2 antibody was not suitable for immunoprecipitation experiments to confirm phosphorylation of the cytosolic tyrosines, we did mutate the residues to phenylalanines to block auto- or trans-phosphorylation.

*VCAM1*-deficient mouse embryonic hearts displayed thinned compact layer, reduction of intraventricular septum and epicardium formation defects, and the interaction between VCAM1 and integrin α4 was shown to be important for heart development^16,17^. Our finding that *VCAP1X2* mutant heart exhibits a thin ventricular compact layer and epicardium formation defect is similar to the phenotype in *VCAM1-*deficient mice. Our data further suggested that decreased pAKT and pERK levels in *VCAP1X2* mutant heart caused sarcomere disorganization and reduction in cardiomyocyte and epicardial cell proliferation, which then resulted in morphologic and functional defects. In contrast, zebrafish VCAM1 was shown to interact with Integrin α9 rather than Integrin α4 and was essential for lymphatic development^41^.

Tyrosine phosphorylation was shown to be crucial during myofibrillogenesis and embryonic heart development in axolotls^42^. Our results further provide a potential molecular mechanism to explain how tyrosine phosphorylation may affect sarcomere structure and function. Like PECAM1, VCAP1X2 may possesses a nonconventional ITAM motif with two tyrosine residues in the cytoplasmic domain that may provide a scaffold for adaptor molecule signaling^43^. SH2-containing phosphatase may bind to phosphorylated tyrosine together with Gab1 to recruit PI3K docking or activate Ras-Raf-MEK-ERK^44^. Alternatively, SH2-containing PI3K p85 may directly bind to phosphorylated tyrosine^45^. Activated AKT kinase would then regulate expression of sarcomeric modulators (*smyd1b*, *mypn*, *fhl2a*) for proper sarcomere assembly that in turn enhances GATA4 activity by promoting translocation of Ankrd1a/ERK/GATA4 complex to the nucleus and ERK-GATA4 signal transduction, driving cardiomyocyte and epicardium proliferation^31^. In addition to regulating cardiomyocyte and epicardium proliferation, activated AKT also regulates Ca^2+^ cycling by modulating expression of *slc8a1a* and *ryr2b* and activity of the Atp2a2a calcium pump for proper cardiac contractility (Supplementary Fig. S9). A previous study showing that VCAM1 activated PI3K and ERK kinase in human airway smooth muscle cells^46^ provides additional support for our finding that VCAP1X2 enhances pAKT and pERK levels.

In conclusion, using a zebrafish gene trap mutant, we demonstrate that VCAP1X2, a cell adhesion molecule, localizes to the myocardium sarcolemma, where it regulates cardiac contractility, and proliferation of cardiomyocytes and epicardial cells by modulating pAKT and pERK expression via two tyrosine residues within the cytoplasmic domain. These results advance our understanding of signal transmission between the sarcolemma and sarcomeres in cardiac muscle cells.

## Methods

### Zebrafish strains and maintenance

Adult zebrafish, including wild-type ASAB, *Tg (myl7: EGFP; myl7: H2AFZ mcherry)*^*cy1*^, *Tg(tcf21:NTR; tcf21:nucEGFP)*, *Tg(tcf21:nucEGFP)*, *Tg(fli1:GFP)* and *VCAP1X2* mutant, were maintained in 20 L aquaria supplied with filtered fresh water and aeration, or high density, self-circulation systems (Aqua Blue) with a photoperiod of 14 h light/10 h dark cycle. Embryos were raised at 28.5 °C and embryonic stages were determined by morphological criteria defined as described^47^. All animal procedures were approved by the Academia Sinica Institutional Animal Care & Utilization Committee (AS IACUC) (protocol # 12-12-482). All experimental methods were performed in accordance with the approved guideline.

### Generation of *VCAP1X2* mutant line

The Tol2 transposon based SAGVG (splice acceptor-Gal4-VP16;UAS:eGFP) plasmid DNA and Tol2 transposase RNA were injected into ASAB 1–2 cell zygotes^19,48,49^. Injected F0 embryos were raised to adulthood and outcrossed with WT to produce F1. F1 Embryos that exhibited intense heart-specific EGFP expression by fluorescence microscopy were raised to adulthood and the F2 generation was produced. Subsequent ligation mediated-PCR showed that the Gal4-VP16; UAS:EGFP vector had been inserted into intron 1 of the *VCAP1X2* gene. To determine whether the splicing of *VCAP1X2* exon 1 and exon 2 was disrupted, RT-PCR was conducted using forward (5′- GATCTACAACAGTGACAGGACA-3′) and reverse (5′-TGTAAATAGTGACTGGGAGAG-3′) primers, and total RNA isolated at 24 and 48 hpf. Homozygous *VCAP1X2* mutant embryos are able to survive to adulthood. Homozygous populations of adult female and male *VCAP1X2* mutants were maintained.

### Morpholino and mRNA injection and RNA synthesis

Individual MOs (3.3 ng/embryo) were diluted with DEPC dH_2_O and injected into randomly selected 1–2 cell zygotes using an IM-300 microinjector (NARISHIGE). For rescue experiments, 33 pg of individual capped *VCAP1X2* mRNA variants together with or without *VCAP1X2* spMO were injected into randomly selected 1–2 cell zygotes of *Tg (myl7: EGFP; myl7: H2AFZ mcherry)* transgenic fish or *VCAP1X2* mutant. For *smyd1b* rescue experiments, 50 pg of capped *smyd1b*^*wt*^, *smyd1b*^*Y247F*^ or s*myd1b*^*278del*^ mRNA was injected into randomly selected 1–2 cell zygotes of *VCAP1X2* homozygous mutant embryos. For overexpression, 66 pg of individual capped *VCAP1X2* mRNA variants were injected into randomly selected 1–2 cell zygotes of *Tg (myl7: EGFP; myl7: H2AFZ mcherry)* transgenic fish.

To synthesize sense mRNA for rescue experiments, *VCAP1X2* variants in the T7TS vector were linearized by SalI digestion and used as template to synthesize capped mRNA by the T7 mMESSAGE mMACHINE Kit (Thermo Fisher Scientific). Myc-DDK tagged *Homo sapiens* vascular cell adhesion molecule 1 (hVCAM1), transcript variant 3 (NM_001199834) plasmid (OriGene Technologies) was linearized by FseI digestion and used to synthesize *hVCAM1* mRNA by a T7 mMESSAGE mMACHINE Kit. To synthesize sense RNA for rescue experiment, *smyd1b*^*wt*^, *smyd1b*^*Y247F*^ or s*myd1b*^*278del*^ mRNA were generated by a SP6 mMESSAGE mMACHINE Kit by digestion with NotI. To synthesize RNA probes for RNA *in situ* hybridization, plasmids were linearized by restriction enzymes and antisense RNA probes were produced as follows (restriction sites and promoters in parentheses): *myl7* (NcoI/sp6), *tcf21*(NcoI/sp6) and *VCAP1X2* (NcoI/sp6).

### Analysis of heart function

72 hpf Zebrafish larvae were anesthetized with a mixture of tricaine (100 ppm) and isoflurane (100 ppm). Green fluorescent images of *Tg(myl7: EGFP; myl7: H2AFZ mcherry)* and *VCAP1X2* mutant hearts were captured as described^20^. Pseudodynamic 3D imaging of the contracting heart was used to measure cardiac function, and the images were constructed and displayed with commercial software (Imaris, Bitplane). End-diastolic volume (EDV) and end-systolic volume (ESV) correspond to the ventricular volume immediately before a contraction and at the end of the contraction, respectively. Stroke volume (SV), the volume of blood ejected in a single heartbeat, was determined from the equation: *SV* = *EDV* - *ESV*, and ejection fraction (EF) was defined as *EF* = *SV*/*EDV*. The FS of zebrafish embryonic hearts was determined as described^50^. In brief, heart beating was recorded at 37 frames per second for 30 s with a camera (AxioCam HRC) installed on a microscope (Zeiss Imager M1). Videos were converted to frames and the ventricular widths and lengths were measured from single frames showing the maximum ventricular systole (VS) and ventricular diastole (VD) by Image J software. FS (%) of the ventricle was calculated as (*Width*_*VD*_ − *Width*_*VS*_)/*Width*_*VD*_ × 100%.

### Immunofluorescence staining

Immunofluorescence staining of zebrafish embryos was performed as described with some modifications^51,52^. For immunostaining of α-actinin, pERK or pAKT, antigen retrieval was conducted by incubating embryos with 150 mM Tris-HCl (pH 9.0) for 15 min at 70 °C. Embryos were then permeabilized by 10 μg/mL proteinase K for 20 min at 28 °C and acetone for 20 min at −20 °C. Embryonic hearts were then dissected using two 22-gauge needles and transferred to slides. Tissues were blocked with 10% serum for 1 h at room temperature (RT), followed by incubation with primary antibody including α-actinin (1:100, Sigma-Aldrich), pAKT (1:100, Cell Signaling) or pERK (1:100, Cell Signaling) at 4 °C overnight. For immunostaining of VCAP1X2 (1:100), embryos were permeabilized in acetone for 7 min at −20 °C and 2% Triton-X100 in PBS for 1.5 h at RT, followed by antigen retrieval in 150 mM Tris-HCl (pH 9.0) for 15 min at 70 °C. For immunostaining of MEF2 or PCNA, embryos were permeabilized by 2% Triton-X100 in PBS for 1.5 h at RT, followed by antigen retrieval in 150 mM Tris-HCl (pH 9.0) for 15 min at 70 °C. Embryonic hearts were then dissected and blocked with 10% serum. Tissues were incubated with primary antibody including MEF2 (1:150, Santa Cruz Biotechnology) or PCNA (1:200, Abcam) at 4 °C overnight. After several PBST washes, embryonic hearts were incubated with goat anti-rabbit Alexa Fluor 647 (1:200, Invitrogen) or mouse Alexa Fluor 568 (1:200, Invitrogen) and later were mounted in ProLong Gold Antifade Mountant with DAPI (Invitrogen). To conduct immunostaining of VCAP1X2 on *Tg(fli1:GFP)* transgenic embryos, fixed *Tg(fli1:GFP)* embryos were permeablized with 2% Triton-X100 in PBS for 1.5 h at RT, followed by antigen retrieval in 150 mM Tris-HCl (pH 9.0) for 15 min at 70 °C and then acetone treatment for 8 min at −20 °C. After several PBST washes, embryos were blocked with 10% serum for 1 h at RT and incubated with anti-VCAP1X2 antibody (1:100) at 4 °C overnight. After several PBST washes, embryos were incubated with goat anti-rabbit Alexa Fluor 647 (1:200, Invitrogen).

### BrdU incorporation

BrdU incorporation was conducted as described^53^. 70.5 hpf embryos were incubated with freshly diluted BrdU (final 10 mM, Sigma-Aldrich) in 15% dimethylsulfoxide on ice for 20 min, and then incubated in egg water at 28.5 °C for 1 h after washes. Samples were fixed in 4% PFA at 72 hpf at 4 °C overnight. Fixed embryos were dehydrated and rehydrated through a methanol/PBST series, followed by permeabilization with 10 µg/mL proteinase K for 30 min and exposure of the BrdU epitope with 2 N HCl for 1 h. Embryos were blocked with 10% serum at 4 °C overnight and incubated with BrdU antibody (1:7, BD Biosciences) at 28 °C for 2 h. After incubation with goat anti mouse Alexa Fluor 647 (1:200, Invitrogen) for 1 h at RT, embryos were washed with PBST before imaging.

### Apoptosis analysis

Embryos were fixed at 72 hpf in 4% PFA at 4 °C overnight. Fixed embryos were dehydrated and rehydrated through a methanol/PBST series, followed by permeabilization with 10 µg/mL proteinase K for 30 min and refixation in 4% PFA for 20 min at RT. Apoptotic cells were labeled by TdT-mediated dUTP nick-end labeling (TUNEL) assay using *in situ* cell death detection kit (Roche), according to the manufacturer’s instructions. After washing with PBST, embryos were blocked with 5% serum in PBST for 2 h at RT, and incubated with anti-fluorescein-POD antibody (1:1000, Roche) and anti-MEF2 antibody (1:150, Santa Cruz Biotechnology) overnight at 4 °C. After several PBST washes, apoptosis signal was enhanced by TSA Plus Cyanine 5 and Flourescein System (Perkin Elmer), then embryos were incubated with goat anti-rabbit Alexa Fluor 568 (1:200, Invitrogen) for 3 h at RT. Later, embryonic hearts were dissected using two 22-gauge needles and transferred to slides, where the tissues were mounted in ProLong Gold Antifade Mountant with DAPI (Invitrogen). Both DNase I-treated positive control and negative control, without TdT enzyme, were included.

### Embryonic heart purification and Western blot analyses

Embryonic heart purification was performed as described^54^. Hearts of 96 hpf *Tg(myl7: EGFP; myl7: H2AFZ mcherry)* transgenic embryos were isolated by fluid force driven by syringe with a 19-gauge needle and filtered through 100 μm and 40 μm cell strainer (Corning). Isolated hearts were washed with L-15 medium containing 10% charcoal-stripped FBS and examined under a fluorescent microscope for intactness. For Western blot analyses, isolated embryonic hearts were extracted by homogenization in 1X RIPA lysis buffer (Millipore) containing 1X Protease Inhibitor Cocktail tablet (Roche), 1X Phosphatase Inhibitor Cocktail 2 and 3 (Sigma-Aldrich) and 1 mM PMSF (Sigma-Aldrich). Protein concentration was determined by Bradford assay. Total protein was separated by electrophoresis on a 11% SDS-PAGE and transferred to Immobilon®-P Transfer Polyvinylidene difluoride (PVDF) Membranes were immunoblotted with total AKT (1:1000), phospho-AKT (1:2000), total ERK (1:2000) or phospho-ERK T202/Y204 (1:2000) antibody from Cell Signaling company, or β-actin (1:10000, Millipore) antibody. Peroxidase-conjugated goat anti-rabbit immunoglobulin G (IgG) (1:5000, Jackson) and peroxidase-conjugated goat anti-mouse IgG (1:5000, Jackson) were used as secondary antibodies. Immuno-reactive signals were detected by Luminata Crescendo Western HRP Substrate (Millipore). Protein concentrations from visualized bands were quantified by Image J.

### Electron Microscopy and sarcomere length quantification

Transmission electron microscopy analysis was conducted as described^55^. For sarcomere length quantification, embryos were incubated with relaxation buffer (20 mM imidazole, 5 mM EGTA, 7 mM MgCl_2,_ 5 mM creatine phosphate (Sigma-Aldrich), 10 mM ATP (Sigma-Aldrich), 100 mM KCl) for 1.5 h before fixation^56^. Z-discs were labelled with anti α-actinin antibody. Sarcomere length and Z-disc length were measured according to immunofluorescent α-actinin signal by Image J.

### Treatment of inhibitors or activator of PI3K or pMEK

To inhibit PI3K-AKT or MAPK-ERK1/2 signaling, *Tg(myl7: EGFP; myl7: H2AFZ mcherry)* embryos were treated with 30 μM PI3K inhibitor (LY294002, Calbiochem) or 40 μM pMEK inhibitor (U0126, Promega) in egg water (final 0.1% DMSO) from 63 to 70.5 hpf, respctively. To activate pAKT, *VCAP1X2* mutant embryos were treated with 1 μM SC-79 (Sigma) in egg water (final 0.1% DMSO) from 63 to 70.5 hpf. These embryos were then replaced with relaxation buffer (final 0.1% DMSO) containing the same amount of inhibitors or activators for 1.5 h before fixation at 72 hpf in 4% PFA overnight at 4 °C. Control embryos were incubated with egg water or relaxation buffer with 0.1% DMSO.

### Microscopy and statistics

Bright-field images of embryos were taken using a camera (AxioCam HRC) installed on a microscope (Zeiss Imager M1) in DIC mode. Immunofluorescence stained embryos or hearts were observed with a confocal microscope (Leica TCS-SP5-MP). Ultrastructural sections were examined using an electron microscope (FEI Tecnai G2 TF20 Super TWIN, Thermo Fisher Scientific). The total number of embryos analyzed (biological replicates) was reported as the sample number for each experiment. Biological replicates were pooled from at least three separate experiments, conducted independently. For RT-qPCR analysis, each biological replicate was analyzed with three technical replications. Two-tailed Student’s *t*-test with unequal variance was performed in Microsoft Excel. ANOVA with *post hoc* multiple comparisons (Bonferroni method) was performed in R^57^.

Plasmid construction, morpholino sequence, VCAP1X2 antibody generation and purification, histological analyses, RNA *in situ* hybridization, reverse transcription polymerase chain reaction (RT-PCR) and quantitative real-time reverse-transcription PCR (RT-qPCR) were performed as described in the Supplementary Information.

### Data availability

All data analyzed during this study are included in this published article (and its Supplementary Information files).

## Electronic supplementary material

Supplementary Information
