# Supplementary material for: Zebrafish VCAP1X2 regulates cardiac contractility and proliferation of cardiomyocytes and epicardial cells
Source: Sci Rep. 2018 May 18;8:7856. doi: 10.1038/s41598-018-26110-3 (PMC5959901; doi:10.1038/s41598-018-26110-3)
Supplement: Supplementary file 1 — Supplementary Information [file 41598_2018_26110_MOESM1_ESM.doc]

**Supplementary Information**

**Zebrafish VCAP1X2 regulates cardiac contractility and proliferation of cardiomyocytes and epicardial cells**

Fang-Chi Hsieh^1^, Yu-Fen Lu^2^, Ian Liau^3^, Chien-Chang Chen^4^, Chao-Min Cheng^5^, Chung-Der Hsiao^6^, Sheng-Ping L. Hwang^1,2,*^

^1^Graduate Institute of Life Sciences, National Defense Medical Center, Taipei 11490, Taiwan

^2^Institute of Cellular and Organismic Biology, Academia Sinica, Taipei 11529, Taiwan

^3^Department of Applied Chemistry and Institute of Molecular Science, National Chiao Tung University, Hsinchu 30010, Taiwan

^4^Institue of Biomedical Sciences, Academia Sinica, Taipei 11529, Taiwan

^5^Institute of Nanoengineering and Microsystems, National Tsing Hua University, Hsinchu 30013, Taiwan

^6^Department of Bioscience Technology, Chung Yuan Christian University, Taoyuan 32023, Taiwan

^*^**For correspondence**: [zoslh@gate.sinica.edu.tw](mailto:zoslh@gate.sinica.edu.tw)

**Supplementary Methods**

**Plasmid construction**

To generate *VCAP1X2* or *tcf21* plasmids for the synthesis of RNA probes for *in situ* hybridization, PCR was conducted using cDNA as template, *VCAP1X2* forward (5′-AGTGACAGGACAATACTAAAGCCC-3′) and reverse (5′- TCACCATTAACTGTGTTTGTGCCG-3′) primers or *tcf21* forward (5′ -CTCCACGTCCAGTCAGAGAACCTC-3′) and reverse (5′ -TATTTGTTTTATTTACAATTATTC-3′) primers, and then the products were cloned into the pGEM-T vector.

To build the *FL* plasmid for the synthesis of full-length *VCAP1X2* mRNA that was used in the rescue experiment, PCR was conducted using cDNA as template, forward (5′-agtagatctACAATACTAAAGCCCAGGACTCG-3′) and reverse (5′-tgatatcTGGGGTCTGTGCTGGCTGCG-3′, restriction sites are underlined) primers. PCR product was cloned into the T7TS vector digested with EcoRV and BglII.

To create *SP* plasmid for the synthesis of *VCAP1X2* exon 1 mRNA, PCR was performed using pT7TS_VCAP1X2 FL plasmid as template, forward (5′-agtagatctACAATACTAAAGCCCAGGACTCG-3′) and reverse (5′- agatatcCTGCAACTAGTGAGAGAAAACAA-3′, restriction sites are underlined) primers, and the product was cloned into the T7TS vector via EcoRV and BglII restriction enzymes.

To produce *Y2F* plasmid for the synthesis of full length *VCAP1X2* mRNA with two cytosolic tyrosines mutated to phenylalanines (Y333F/Y341F), the nucleotide sequence between 235 and 1308 bp was amplified by a first PCR using pT7TS_VCAP1X2 FL plasmid as template and 5′- agtagatctACAATACTAAAGCCCAGGACTCG-3′ and 5′-CTTGAAATTA*a*AATGGCCCATCTTGTT-3′ (restriction site is underlined and mutated nucleotide is lowercase and italicized) primers. The nucleotide sequence between 1288 and 1356 bp was amplified by a second PCR using pT7TS_VCAP1X2 FL plasmid as template and 5′-ATGGGCCATT*t*TAATTTCAAGAAGCC-3′ and 5′-tgatatcTGGGGTCTGTGCTGGCTGCG-3′ (restriction site is underlined and mutated nucleotide is lowercase and italicized) primers. A third PCR was conducted to merge these two fragments, which created the VCAP1X2-Y341F construct. The products of the first and second PCR were used as template with 5′-agtagatctACAATACTAAAGCCCAGGACTCG-3′ and 5′-tgatatcTGGGGTCTGTGCTGGCTGCG-3′ (restriction sites are underlined) primers. The PCR product was cloned into pT7TS vector digested with BglII and EcoRV. Subsequently, the nucleotide sequence between 235 and 1284 bp was amplified in a first PCR using pT7TS_ VCAP1X2-Y341F plasmid as template and 5′- agtagatctACAATACTAAAGCCCAGGACTCG-3′ and 5′- GTTTTTTTTG*a*AGCAGGTGATGTAGAT-3′ (restriction site is underlined and mutated nucleotide is lowercase and italicized) primers. The nucleotide sequence between 1264 and 1356 bp was amplified in a second PCR using pT7TS_ VCAP1X2-Y341F plasmid as template and 5′-ATCACCTGCT*t*CAAAAAAAACAAGATG-3′ and 5′-tgatatcTGGGGTCTGTGCTGGCTGCG-3′ (restriction site is underlined and mutated nucleotide is lowercase and italicized) primers. A third PCR was conducted to merge these two fragments in order to form the VCAP1X2-Y333F/Y341F construct. The products of the first and second PCR were used as template with 5′-agtagatctACAATACTAAAGCCCAGGACTCG-3′ and 5′-tgatatcTGGGGTCTGTGCTGGCTGCG-3′ (restriction sites are underlined) primers and the product was cloned into pT7TS vector via BglII and EcoRV restriction enzymes.

To generate the *△N* plasmid for the synthesis of *VCAP1X2* mRNA lacking extracellular ICAM and Ig domains, but containing signal peptide and two cytosolic tyrosine residues, or *△N Y2F* plasmid for the synthesis of *VCAP1X2* mRNA lacking extracellular ICAM and Ig domains, but containing signal peptide and two cytosolic Y333F/Y341F mutations, the nucleotide sequence between 277 and 456 bp was amplified in a first PCR using pT7TS_VCAP1X2 FL or pT7TS_VCAP1X2-Y333F/Y341F plasmid as template and 5′-tagcggccgcATGCAGAAAGGGTCGTTCTTTC-3′ and (5′-CGATTATTATTGTGACTGAAGTGCTGCAGT-3′) (restriction site is underlined) primers. The nucleotide sequence between 1201 and 1356 bp was amplified in a second PCR using pT7TS_VCAP1X2 FL or pT7TS_VCAP1X2-Y333F/Y341F plasmid as template and 5′-TTCAGTCACAATAATAATCGGCATTGTTTG-3′ and 5′-gtaccggtTGGGGTCTGTGCTGGCTGCG-3′ primers. A third PCR was conducted to merge these two fragments and create either *△N* or *△N Y2F* using the products of the first and second PCR as template and 5′-tagcggccgcATGCAGAAAGGGTCGTTCTTTC-3′ and 5′-gtaccggtTGGGGTCTGTGCTGGCTGCG-3′ (restriction sites are underlined) primers. The PCR product was cloned into the PCS2^+^ vector digested with NotI and AgeI. Later, PCR was conducted using PCS2^+^_△N or PCS2^+^_△N Y2F plasmid as template and 5′-cgcagatctATGCAGAAAGGGTCGTTCTTTC-3′ and 5′-tgatatcTGGGGTCTGTGCTGGCTGCG -3′ (restriction sites are underlined) primers and the product was cloned into T7TS vector digested with BglII and EcoRV.

**Morpholino**

Morpholino antisense oligonucleotides were synthesized by Gene Tools (Philomath, OR, USA). They included *VCAP1X2* 5 mismatched (5mm) spMO (5′ –CTAaTGaACaTaTTAAACAaAATGA-3′) (mismatched nucleotides are lower case) and *VCAP1X2* spMO (5′-CTAGTGCACCTGTTAAACACAATGA-3′) targeted to the acceptor site of *VCAP1X2* intron 1. MOs were individually dissolved in Danieau buffer (58 mM NaCl, 0.7 mM KCl, 0.4 mM MgSO_4_, 0.6 mM Ca(NO_3_)_2_, 5 mM HEPES, pH 7.6) to 1 mM solution as stock.

**VCAP1X2 antibody generation and purification**For VCAP1X2 antigen epitope preparation, the cDNA sequence coding the extracellular domain without the signal peptide (31-308 amino acids) was amplified using forward primer (5′-CAGGTACCGCACTAGAATGTCCTCTTCAAATC-3′) and reverse primer (5′-AGAAGCTT*TCA*AGGCGTTATATTCTCTTGAATGAC-3′) (restriction enzyme site is underlined and stop codon is italicized) and Phusion DNA polymerase. Amplified DNA was digested with KpnI and HindIII and cloned into pQE30 vector (Qiagen, GmbH, Germany). Induced recombinant protein was found in the insoluble fraction and purified with Ni-NTA agarose resin (QIAGEN) under denaturing conditions. The purified recombinant protein was provided to LTK BioLaboratories (Taoyuan, Taiwan) and used as antigen to generate anti-VCAP1X2 antibody in rabbit. Boosted antibody was then purified from rabbit serum by NHS-activated Sepharose 4 Fast Flow (GE Healthcare, Buckinghamshire, UK).

**Histological analyses**Embryos at 120 hpf were fixed with 4 % paraformaldehyde (PFA) in PBS at 4 °C overnight. After removing residual PFA, embryos were dehydrated with a series of graded ethanol and then infiltrated in wax. The wax filtrated embryos were embedded into paraffin blocks for 5 μm serial sectioning. After slides were deparaffinized and rehydrated, sections were stained with hematoxylin and eosin.

**RNA *in situ* hybridization**

Embryos were raised in egg water with 0.003% phenylthiourea and fixed with 4% PFA at 4 °C overnight. After dehydration and rehydration in a series of methanol/PBS with 0.1% tween 20 (PBST) solutions, RNA *in situ* hybridization was performed on embryos using digoxigenin-labeled antisense RNA probes and alkaline phosphatase-conjugated anti-digoxigenin antibodies as previously described ^1^.

**Reverse transcription polymerase chain reaction (RT-PCR) and quantitative real-time reverse-transcription PCR (RT-qPCR)**

To evaluate the transcript expression level of *VCAP1X1* and *VCAP1X2*, RT-PCR was conducted using cDNA from 72 and 96 hpf embryos as template, and forward (5′-CGGATGTCCCATATAGGACTACT-3′) primer and reverse (5′-ATTTTCCTCTGGTCCACAGCATG-3′) primer for *VCAP1X1* and reverse (5’-AGTTGGAGTAATGAGGTGTTTAATA-3’) primer for *VCAP1X2*, and forward (5’-CCATTGGCAATGAGAGGTTCAG-3’) and reverse (5’TGATGGAGTTGAAAGTGGTCTCG-3’) primers for β-actin.

To evaluate the efficacy of spMO, RT-PCR was conducted using cDNA from 26 hpf embryos as template, and forward (5′-ACCTGCTTTTCATTGTGTTTAACAG-3′) and reverse (5′-TGGATGTCCTTCCGATTTACAA-3′) primers. To perform RT-qPCR analyses, total RNA was extracted from isolated embryonic heart as previously described ^2^. RT-qPCR was performed by addition of 20 ng cDNA, forward and reverse primers (5 pmole) for target genes and 2x SYBR Green I master mix (Roche) using a Roche Light Cycler 480 II thermal cycler. The PCR program was set 95 °C 10 min for 1 cycle, 95 °C 10 sec, 60 °C 10 sec, 72 °C 10 sec for 45 cycles. The gene specific primers are listed in Supplementary Table S1.

**Supplementary References**

1 Thisse, C. & Thisse, B. High-resolution in situ hybridization to whole-mount zebrafish embryos. *Nat Protoc* **3**, 59-69, doi:10.1038/nprot.2007.514 (2008).

2 Lombardo, V. A., Otten, C. & Abdelilah-Seyfried, S. Large-scale zebrafish embryonic heart dissection for transcriptional analysis. *J Vis Exp*, 52087, doi:10.3791/52087 (2015).

**Supplementary Figures and Legends**


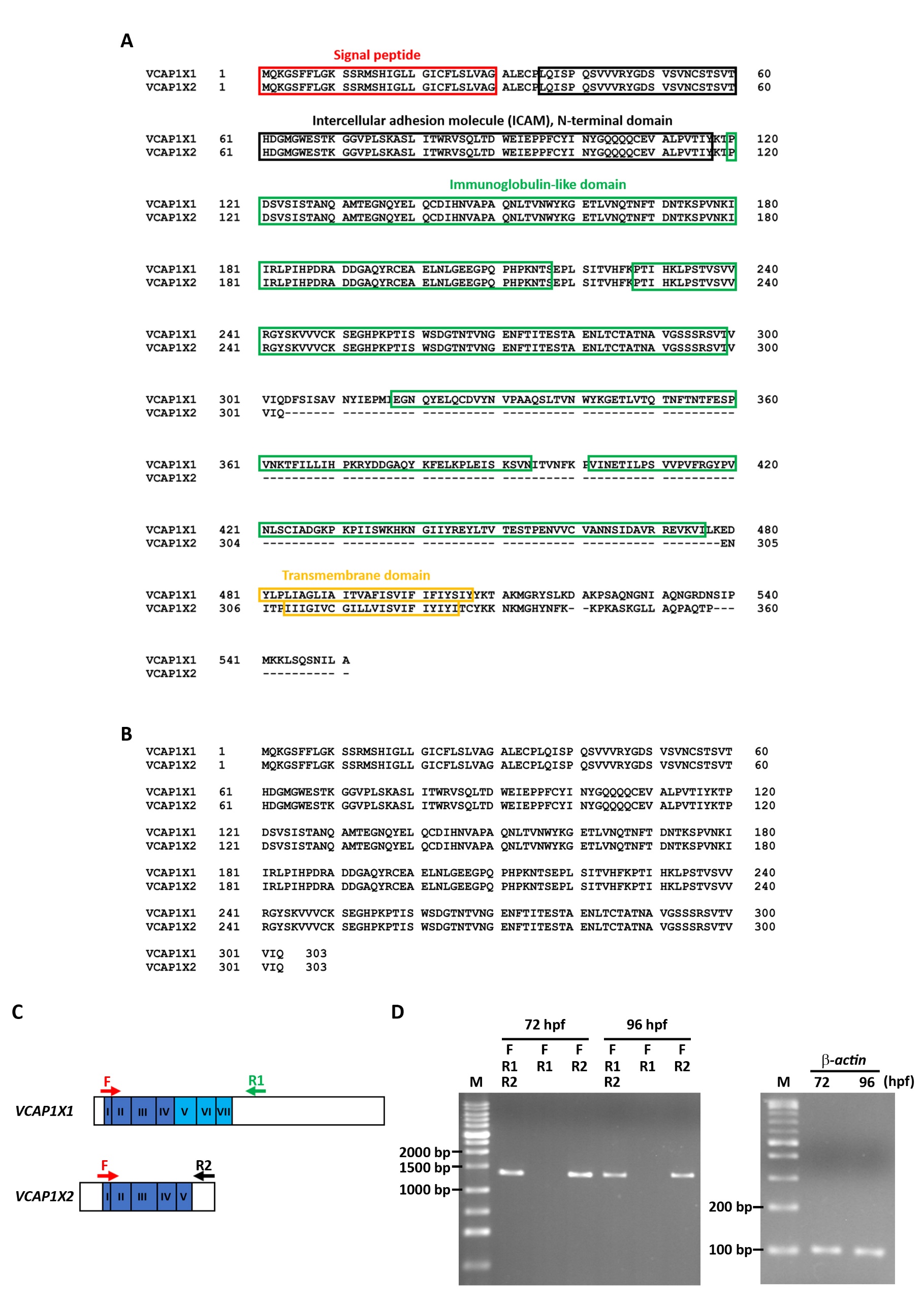


**Figure S1.** Comparison of protein domains, amino acid sequences and expression levels of *VCAP1X1* and *VCAP1X2* genes. (**A**) VCAP1X2 protein contains a signal peptide, one intercellular adhesion molecule (ICAM) domain, two immunoglobulin (Ig) superfamily domains, a transmembrane domain, and a cytosolic domain while VCAP1X1 protein possesses extra two Ig domains. (**B**) VCAP1X1 and VCAP1X2 are identical between amino acid numbers 1-303. (**C**) A diagram shows the location of forward (F) primer, *VCAP1X1* specific reverse (R1) primer, and *VCAP1X2* specific reverse (R2) primer. (**D**) RT-PCR showed the presence of 1361 bp DNA band reaction containing F, R1 and R2 primers or F and R2 primers but not in a reaction containing F and R1 primers. DNase I-treated total RNA isolated from 72 or 96 hpf embryos was used in the reverse transcription reaction and *β-actin* served as a control. M, molecular weight marker.


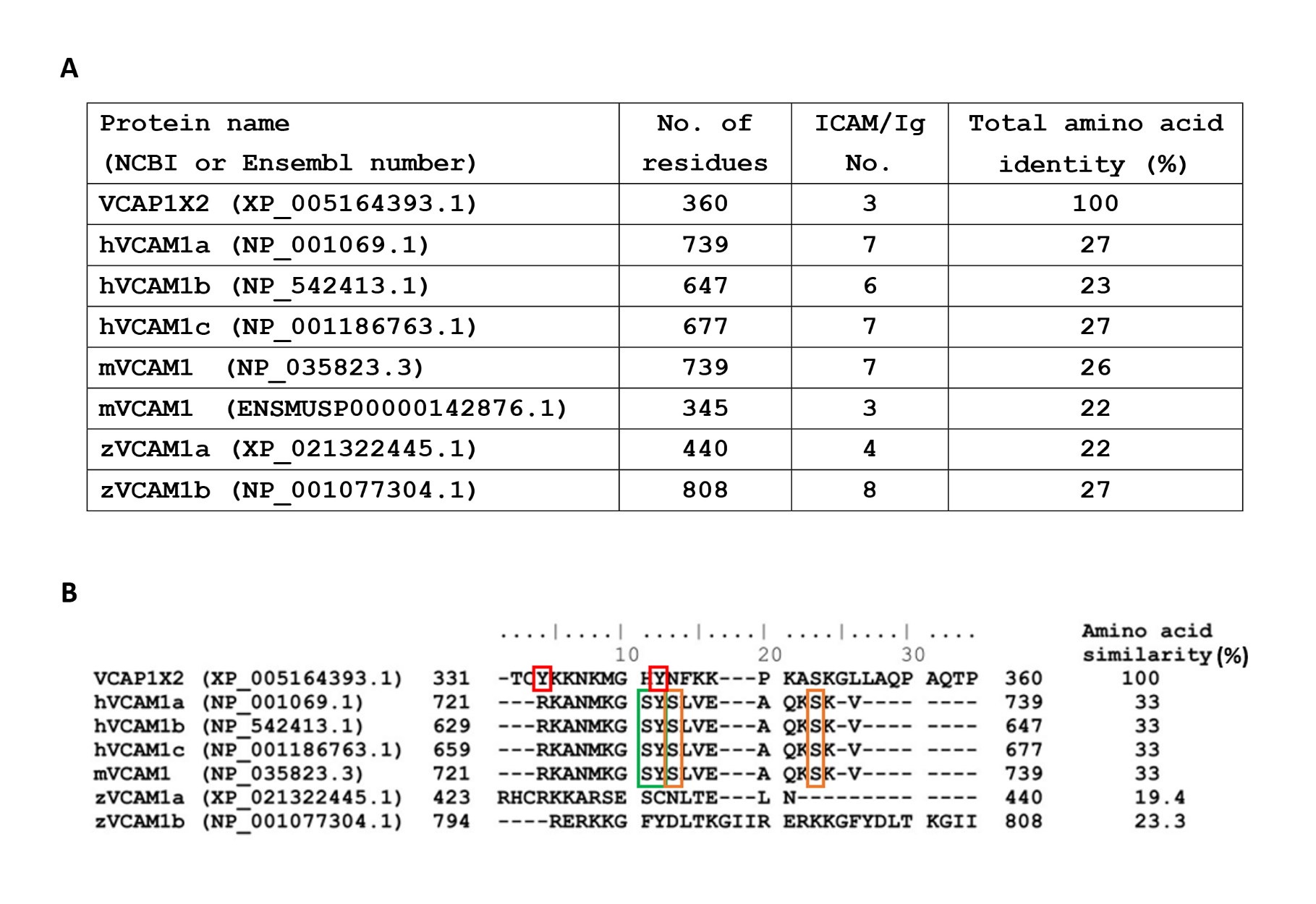


**Figure S2.** Amino acid comparison between VCAP1X2 and VCAM1 isoforms from human, mouse and zebrafish. (**A**) Total amino acid number, number of intercellular adhesion molecule (ICAM) or immunoglobulin-like (Ig) domains, and percentage of total amino acid identity between VCAP1X2 and different VCAM1 isoforms from human, mouse and zebrafish are shown. (**B**) The amino acid sequences within the cytoplasmic region and amino acid similarity among VCAP1X2 and different VCAM1 isoforms from human, mouse and zebrafish were compared. Serine and tyrosine residues (boxed in green) are necessary for mammalian VCAM1 activation of calcium flux. Two serine residues (boxed in orange) are required for VCAM1 activation of Rac1 during leukocyte transendothelial migration. Two cytosolic tyrosine residues (boxed in red) for VCAP1X2 function are indicated.


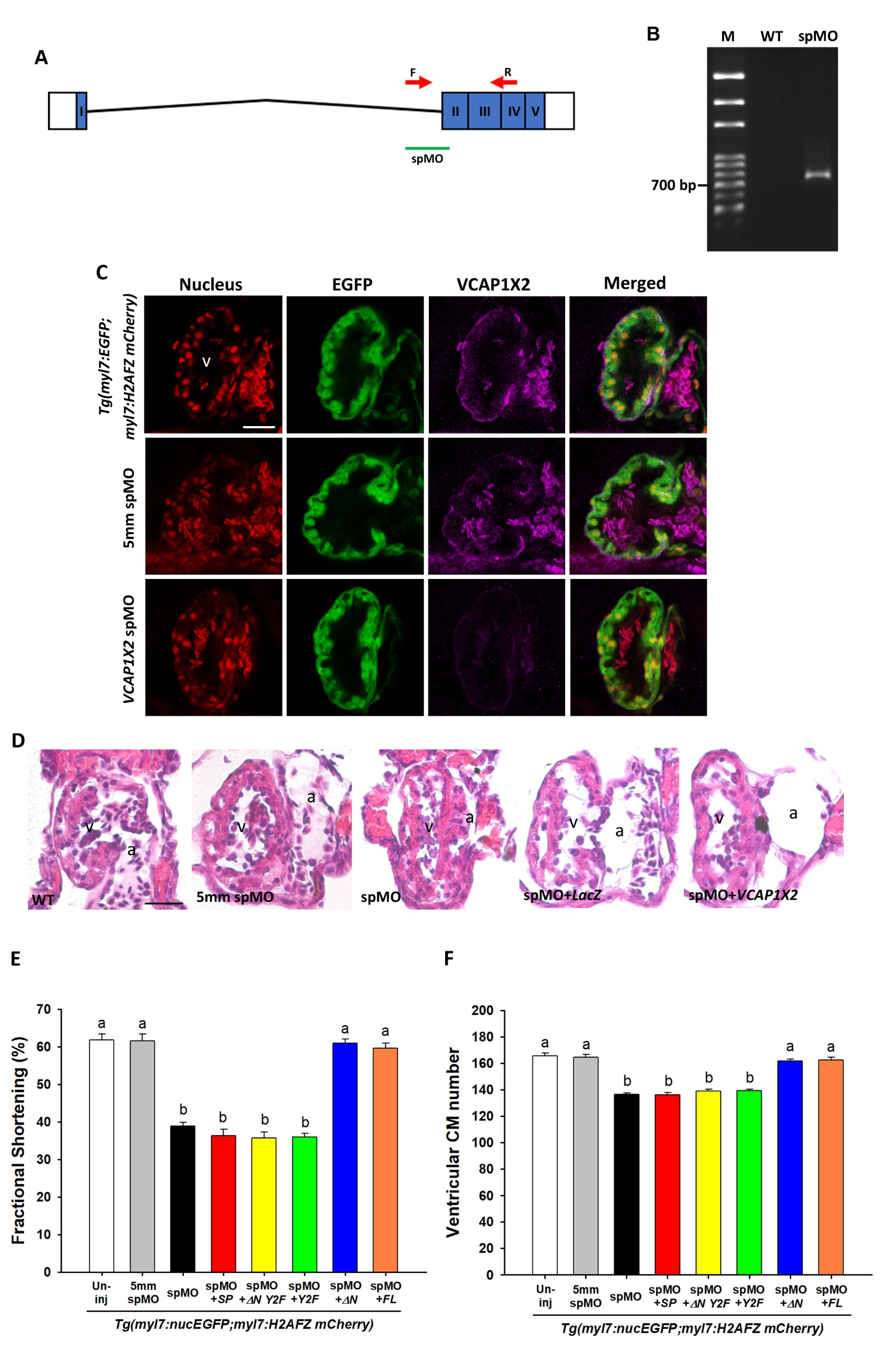


**Figure S3.** *VCAP1X2* morphant embryos exhibited similar cardiac defects to *VCAP1X2* mutant embryos. (**A**) A diagram of *VCAP1X2* spMO target site at the boundary between intron 1 and exon 2 is shown. (**B**) Splicing of exon 1 and exon 2 was disrupted in *VCAP1X2* morphant compared to wild-type (WT) embryos, indicated by the 702 bp RT-PCR product using forward and reverse primers indicated by red arrows in (A). (**C**) Expression of EGFP or VCAP1X2 in ventricles of hearts in *Tg*(*myl7: EGFP; myl7: H2AFZ mcherry*), 5mm spMO-injected, and *VCAP1X2* spMO-injected embryos. Higher VCAP1X2 expression was detected in the heart ventricle in *Tg* (*myl7: EGFP; myl7: H2AFZ mcherry*) embryos and 5mm spMO-injected embryos. No VCAP1X2 expression was detected in the *VCAP1X2* spMO-injected embryos at 96 hpf (n = 15 per condition, N = 3). v, ventricle. Scale bar, 30 μm. (**D**) Paraffin sections stained with hematoxylin and eosin showed thinner ventricular compact layer in *VCAP1X2* spMO-injected morphants compared to un-injected wild-type (WT) or 5mm spMO-injected control embryos at 120 hpf. Such defects could be rescued by co-injection with full-length (*FL*) *VCAP1X2* but not with *LacZ* mRNA (n = 10 per condition). Scale bar, 25 μm. v, ventricle, a, atrium. (**E**) *VCAP1X2* spMO-injected *Tg(myl7:EGFP; myl7:H2AFZ mCherry)* embryos exhibited decreased ventricular fractional shortening in the heart compared to un-injected or 5mm spMO-injected transgenic embryos and co-injection of *△N* or full-length (*FL*) *VCAP1X2* mRNA, but not *SP*, *△N Y2F* or *Y2F* *VCAP1X2* mRNA, could rescue decreased ventricular fractional shortening in the heart of *VCAP1X2* spMO-injected transgenic embryos at 72 hpf (n = 20 per condition, N = 3). Error bars indicate standard error. (**F**) *VCAP1X2* spMO-injected *Tg(myl7:EGFP; myl7:H2AFZ mCherry)* embryos exhibited reduced ventricular cardiomyocyte (CM) number compared to uninjected or 5mm spMO-injected transgenic embryos at 72 hpf. The defect could be rescued by co-injection of *△N* or full-length (*FL*) *VCAP1X2* mRNA, but not *SP*, *△N Y2F* or *Y2F* *VCAP1X2* mRNA (n = 20 per condition, N = 3). Error bars indicate standard error. Quantitative data were analyzed by ANOVA with Bonferroni multiple comparisons. Treatments that are not statistically different (α = 0.05) from each other are labeled with the same letter.


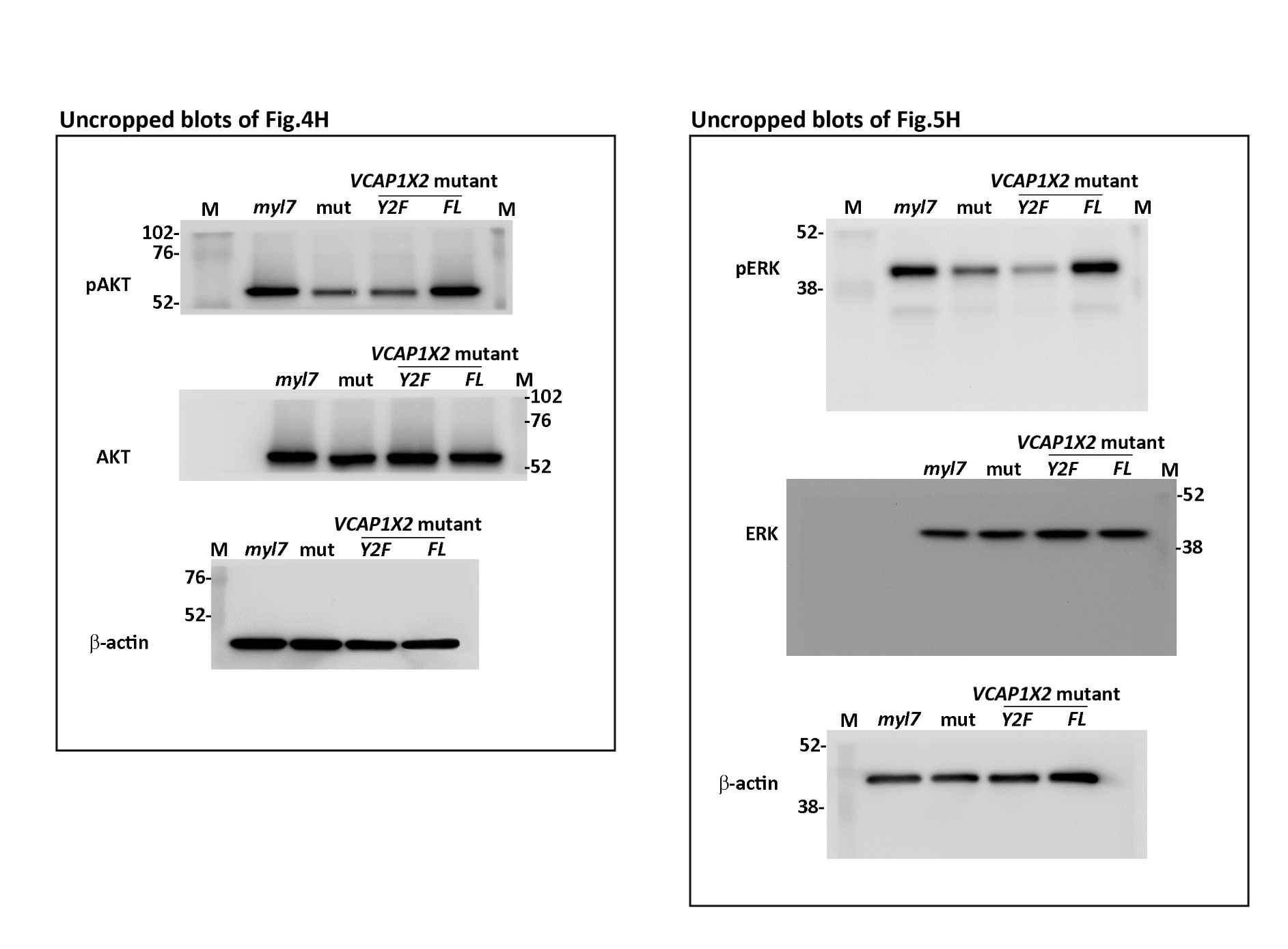


**Figure S4.** Representative uncropped blots for evaluation of pAKT or pERK expression levels in *Tg*(*myl7:EGFP; myl7:H2AFZ mCherry*), *VCAP1X2* mutant or *VCAP1X2* mutants injected with *Y2F* or full-length *VCAP1X2* mRNA. Levels of pAKT or total AKT in embryonic hearts isolated from *Tg*(*myl7:EGFP; myl7:H2AFZ mCherry*) (*myl7*), *VCAP1X2* mutant (mut) or *VCAP1X2* mutant injected with *Y2F* or full-length (*FL*) *VCAP1X2* mRNA were determined by Western blot with β-actin as loading control. The size (kDa) of different marker proteins is indicated.


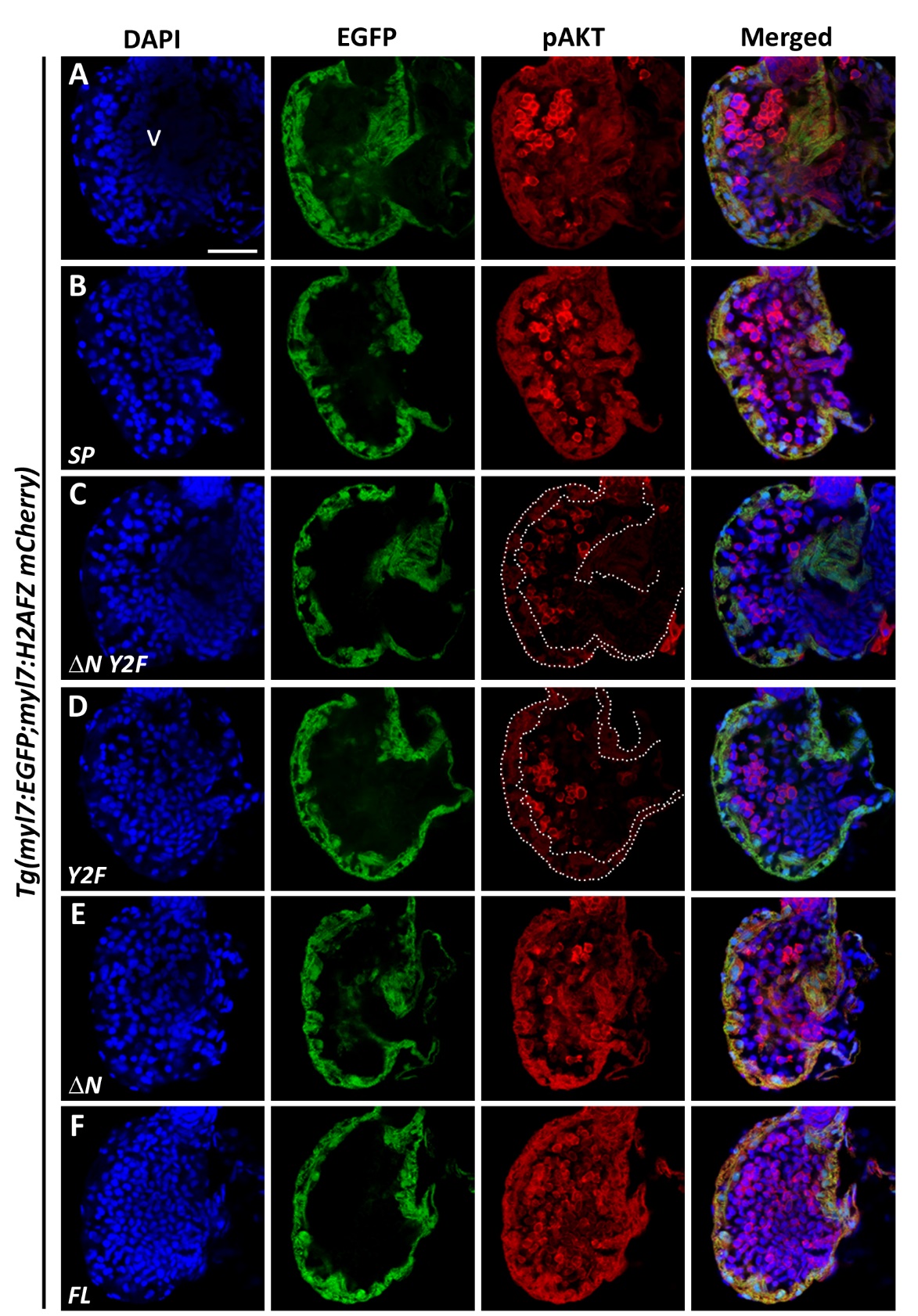


**Figure S5.** Endogenous expression of pAKT was nearly abolished in heart ventricles of *△N Y2F* or *Y2F* *VCAP1X2* mRNA-overexpressing *Tg(myl7:EGFP; myl7:H2AFZ mCherry)* embryos. Decreased expression level of pAKT was detected in heart ventricles of *Tg(myl7:EGFP; myl7:H2AFZ mCherry)* embryos that overexpressed *△N Y2F* (C) or *Y2F VCAP1X2* (D) mRNA compared to un-injected (A) or transgenic embryos that overexpressed *SP* (B), *△N* (E) or *FL* (F) *VCAP1X2* mRNA at 96 hpf (n = 15 per condition, N = 3). Dashed lines indicate the ventricular myocardium region. Erythrocytes in the ventricular chamber show non-specific staining from secondary antibody. Nuclei were labeled by DAPI staining. v, ventricle. Scale bar, 30 μm.


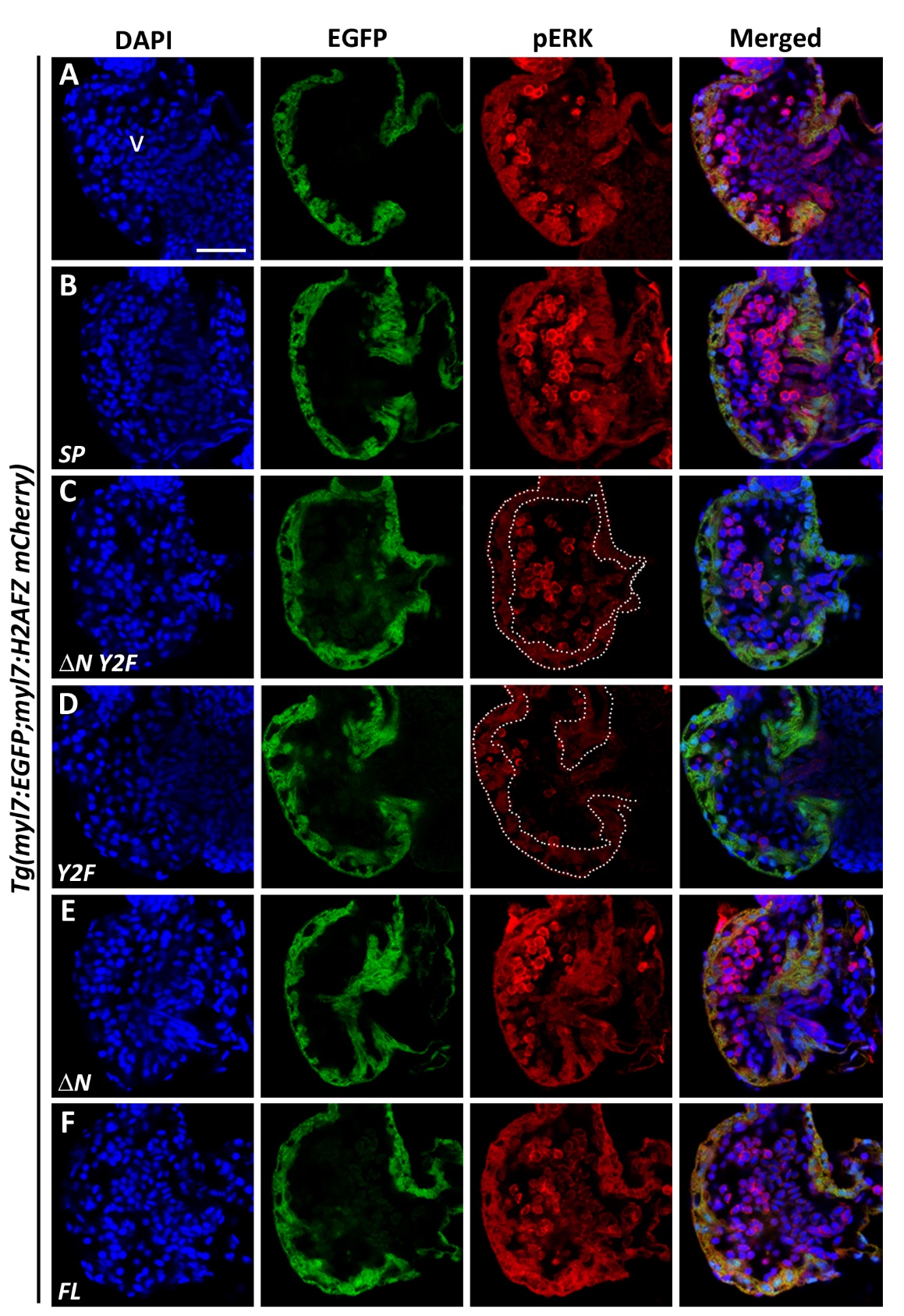


**Figure S6.** Endogenous expression of pERK was nearly abolished in heart ventricles of *△N Y2F* or *Y2F* *VCAP1X2* mRNA-overexpressing *Tg(myl7:EGFP; myl7:H2AFZ mCherry)* embryos. Decreased expression level of pERK was detected in heart ventricles of *Tg(myl7:EGFP; myl7:H2AFZ mCherry)* embryos that overexpressed *△N Y2F* (C) or *Y2F VCAP1X2* (D) mRNA compared to un-injected (A) or transgenic embryos that overexpressed *SP* (B), *△N* (E) or *FL* (F) *VCAP1X2* mRNA at 96 hpf (n = 15 per condition, N = 3). Dashed lines indicate the ventricular myocardium region. Erythrocytes in the ventricular chamber show non-specific staining from secondary antibody. Nuclei were labeled by DAPI staining. v, ventricle. Scale bar, 30 μm.


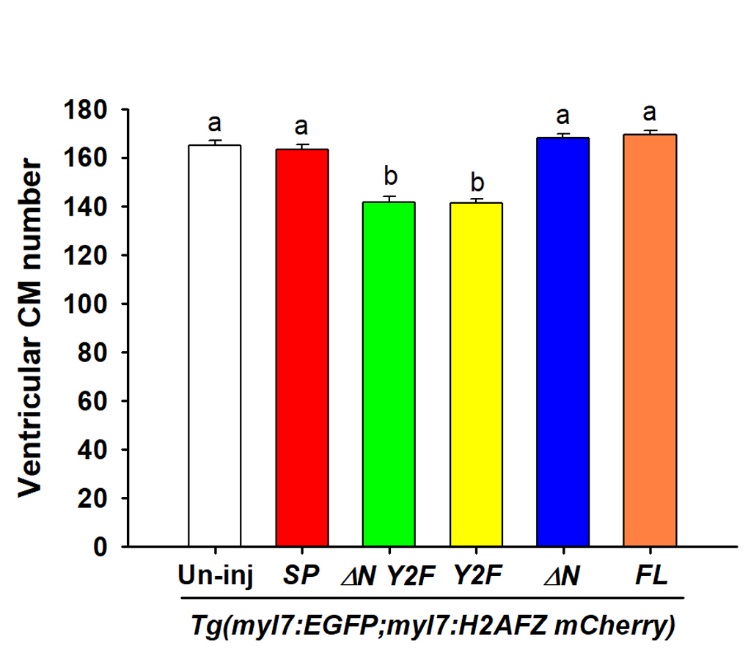


**Figure S7.** Reduced ventricular cardiomyocyte number was observed in heart ventricles of *△N Y2F* or *Y2F VCAP1X2* mRNA-overexpressing *Tg(myl7:EGFP; myl7:H2AFZ mCherry)* embryos. Compared to un-injected or transgenic embryos that overexpressed *SP*, *△N* or full-length (*FL*) *VCAP1X2* mRNA, decreased ventricular cardiomyocyte (CM) number was observed in *△N Y2F* or *Y2F VCAP1X2* mRNA-overexpressing *Tg(myl7:EGFP; myl7:H2AFZ mCherry)* embryos at 96 hpf. (n = 20 per condition, N = 3). Error bars indicate standard error. Quantitative data were analyzed by ANOVA with Bonferroni multiple comparisons (α = 0.05). The difference between groups a and b was significant (*p* < 0.001).


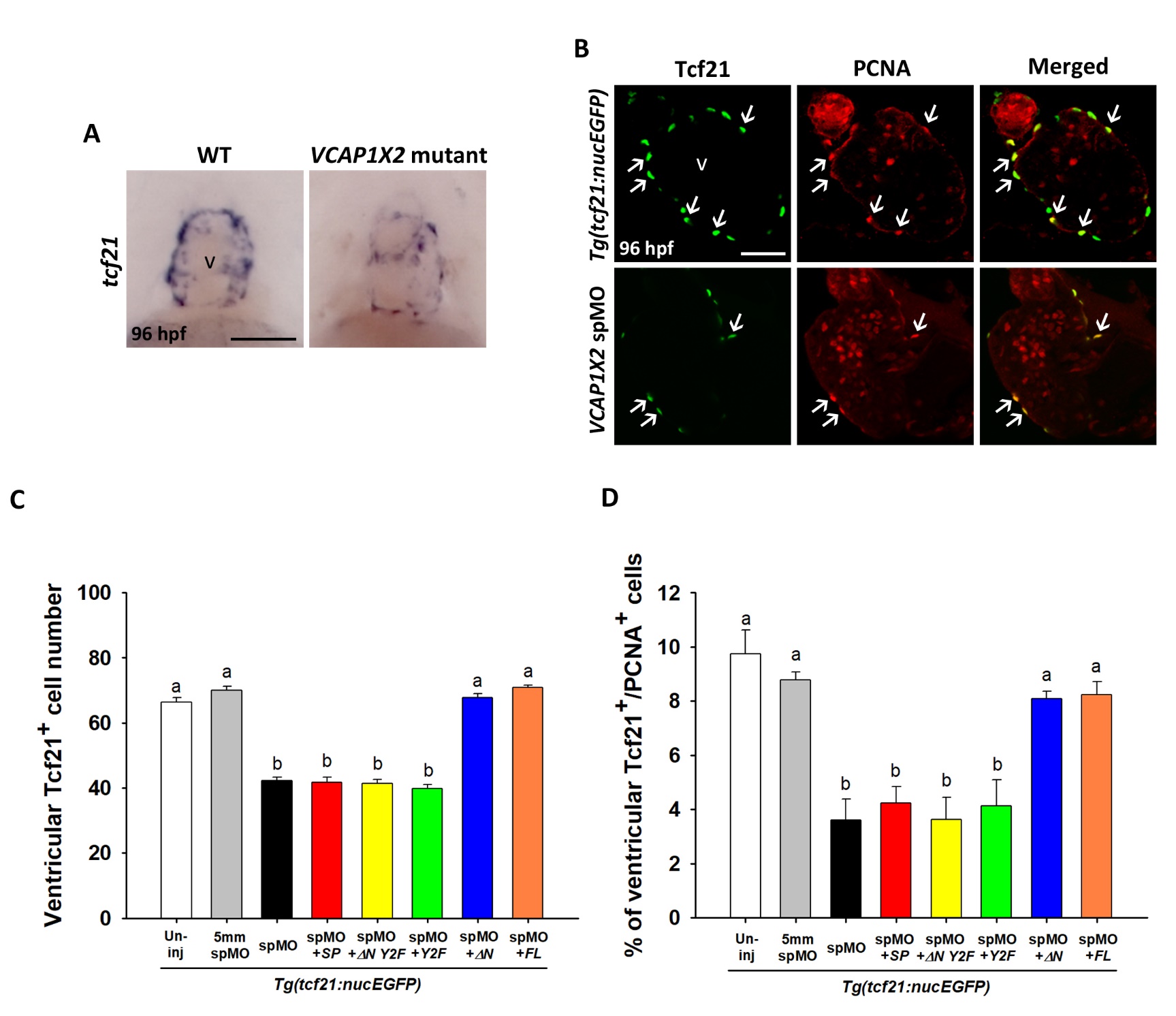


**Figure S8.** Epicardium formation was affected in *VCAP1X2* mutants and morphants, but could be rescued by co-injection of *△N* or full-length (*FL*) *VCAP1X2* mRNA. (**A**) Epicardium defects were detected by *tcf21* staining in the ventricle of *VCAP1X2* mutant, but not wild-type (WT) heart at 96 hpf (n = 15 per condition, N = 3). Scale bar, 50 μm. v, ventricle. (**B**) Tcf21^+^ epicardial cell number was reduced with coincident decreases in PCNA^+^ cell number in *VCAP1X2* spMO-injected *Tg(tcf21:nucEGFP)* transgenic embryos compared to un-injected transgenic embryos at 96 hpf. Arrows indicate Tcf21^+^/PCNA^+^ epicardial cells. Scale bar, 25 μm. (**C**) Quantification of Tcf21^+^ epicardial cell number in ventricles of *Tg(tcf21:nucEGFP)* transgenic embryos was performed following different treatments (n = 20 per condition, N = 3). (**D**) The percentage of PCNA^+^ proliferative epicardial cells in ventricles of *Tg(tcf21:nucEGFP)* transgenic embryos following different treatments was compared (n = 20 per condition, N = 3). Error bars indicate standard error. Quantitative data were analyzed by ANOVA with Bonferroni multiple comparisons. Treatments that are not statistically different (α = 0.05) from each other are labeled with the same letter.

**
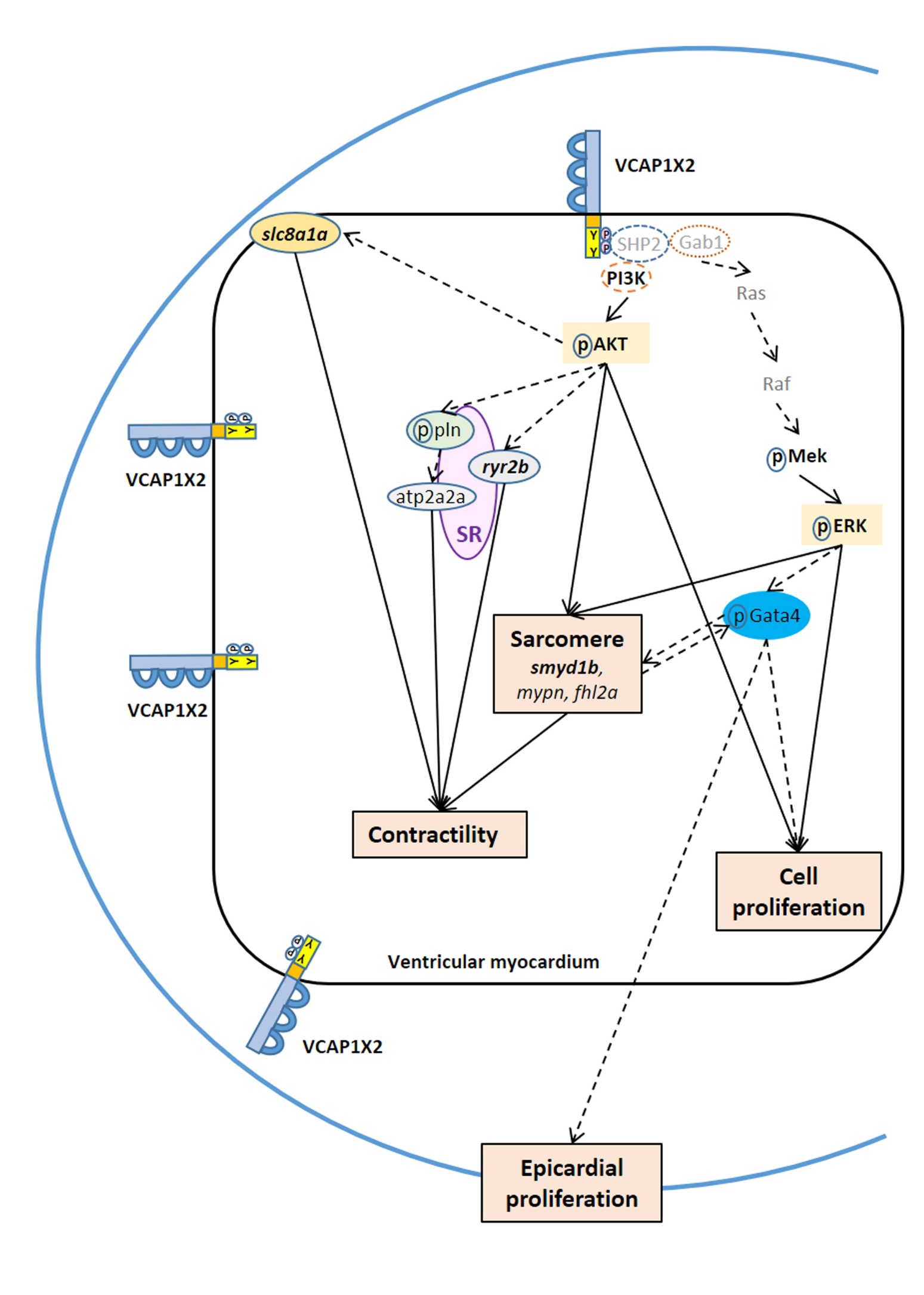
**

**Figure S9.** Proposed model for VCAP1X2 function in regulating cardiac contractility and proliferation of cardiomyocytes and epicardial cells. The hypothetical association of SHP2, Gab1 or PI3K with phosphotyrosines in the cytosolic region of VCAP1X2 remains to be investigated.

| **Supplementary Table S1.** Real time-qPCR primer list | |  |
| --- | --- | --- |
| **Gene** | **Forward (5’-3’)** | **Reverse (5’-3’)** |
| ankrd1a | TAAAGGTGTCTCGCGCTGTT | TCCCCAAGTGATCGGTGTTG |
| ankrd1b | GGAGGTAGTTTGCCTGTGCT | GCGTGTCACCCTCTATGTCT |
| atp2a2a | ATCGGTAAGATCCGTGATGA | CACGGCGATCTTGAAGTAGT |
| b-actin | CCATTGGCAATGAGAGGTTCAG | TGATGGAGTTGAAAGTGGTCTCG |
| bmp4 | GGAGTGCTGTTCTCGAGTGT | ACGGGTCTCAAAGGAGTTCG |
| cryabb | CCCCGTAACTCGTGATGACA | CACAAACATTTGGCCGTCAGT |
| desma | ATCAGAGATCCCGTGTGGAG | CCCTCGATACGTCTTTCCAG |
| fhl2a | TGCTGTGTACCGAGTGCTAC | AGGGCACGCAGTAGTTATGG |
| gata4 | CGCTCGTGGAGCAATAATCG | CTGGATCATCGGAGTCACCC |
| hsp90aa1.1 | AGCCAGACTTCGGTGAATCAA | TTCTCTCTGTTTCTCAATGTAAA |
| itgb1bp2 | CGGACGCCACAGTAATGAGA | CACCTGCAGCTTTGTCTTGG |
| mybpc3 | AGCACTATGACCGCAACACT | TCTGTGCTAGTGCGTCTAAATG |
| myh6 | TGAAGACCTGAGAAGGCAAC | CAGTTCCTCGGTTCTCTGAA |
| myh7 | TCAGATGGCAGAGTTTGGAG | GCTTCCTTTACAGTTACAGTCTTTC |
| mypn | TCCACCAATGAAGAAGAGCAGT | TGTCCTGAAAGGCTGATGGTC |
| nexn | GAAACGTTCCCGGAGGATGA | TGGCCCTACCGTGAGAAATG |
| nppa | GATGTACAAGCGCACACGTT | TCTGATGCCTCTTCTGTTGC |
| nppb | CATGGGTGTTTTAAAGTTTCTCC | CTTCAATATTTGCCGCCTTTAC |
| pln | AAGGTGCAGCACATGACAC | CCGTGGAAAGAGATTAGCAA |
| ryr2b | CGAGACAAAGCTCAGGATGT | TTCGTGAGGGACTCTTTCAG |
| slc8a1a | AGGAGTTTTTAGACGGCGCA | AGGACGTGGTAGTTGGCAAG |
| smyd1b | ATCTGAACGTGTCTGCAGA | TCTTCCGGCACCTTGACTCCATCC |
| tbx5a | GATAGCGCAAGTCCCTCACT | CCCAGCACTAGCGGTTGATA |
| tcap | GGGAGGAAAACCCAAATAAGAG | TGGAGAGGACGCACCTGCCA |
| tnnt2a | CAATGTCCTGAGGAACAGAGTCA | TTTCAACAGTGGTCAGCTCC |
| unc45b | GCTGCAAGGAGGTCCAAGACA | GATCATCAGCATCCAGCATGT |
| vegfaa | CTGTAATGATGAGGCGCTCG | AGGCTCACAGTGGTTTTCTTTC |
| vmhcl | TGTTAAAGCCACCGTCGTGA | TGCTGGCTCATGGAGAAAGG |
